# Direct Delivery of MicroRNA96 to the Lungs Reduces Progression of Sugen/Hypoxia-Induced Pulmonary Hypertension in the Rat

**DOI:** 10.1016/j.omtn.2020.09.002

**Published:** 2020-09-06

**Authors:** Craig K. Docherty, Nina Denver, Simon Fisher, Margaret Nilsen, Dianne Hillyard, Rebecca L. Openshaw, Hicham Labazi, Margaret R. MacLean

**Affiliations:** 1 Strathclyde Institute of Pharmacy and Biological Sciences, University of Strathclyde, Glasgow G4 0RE, Scotland; 2 Institute of Cardiovascular and Medical Sciences, College of Medical Veterinary and Life Sciences, University of Glasgow, Scotland

**Keywords:** pulmonary hypertension, serotonin, microRNA96

## Abstract

The 5HT1B receptor (5HT1BR) contributes to the pathogenic effects of serotonin in pulmonary arterial hypertension. Here, we determine the effect of a microRNA96 (miR96) mimic delivered directly to the lungs on development of severe pulmonary hypertension in rats. Female rats were dosed with sugen (30 mg/kg) and subjected to 3 weeks of hypobaric hypoxia. In normoxia, rats were dosed with either a 5HT1BR antagonist SB216641 (7.5 mg/kg/day for 3 weeks), miR96, or scramble sequence (50 μg per rat), delivered by intratracheal (i.t) administration, once a week for 3 weeks. Cardiac hemodynamics were determined, pulmonary vascular remodeling was assessed, and gene expression was assessed by qRT-PCR, and *in situ* hybridization and protein expression were assessed by western blot and ELISA. miR96 expression was increased in pulmonary arteries and associated with a downregulation of the 5HT1BR protein in the lung. miR96 reduced progression of right ventricular systolic pressure, pulmonary arterial remodeling, right ventricular hypertrophy, and the occurrence of occlusive pulmonary lesions. Importantly, miR96 had no off-target effects and did not affect fibrotic markers of liver and kidney function. In conclusion, direct delivery of miR96 to the lungs was effective, reducing progression of sugen/hypoxia-induced pulmonary hypertension with no measured off-target effects. miR96 may be a novel therapy for pulmonary arterial hypertension, acting through downregulation of 5HT1BR.

## Graphical Abstract

**Figure:**
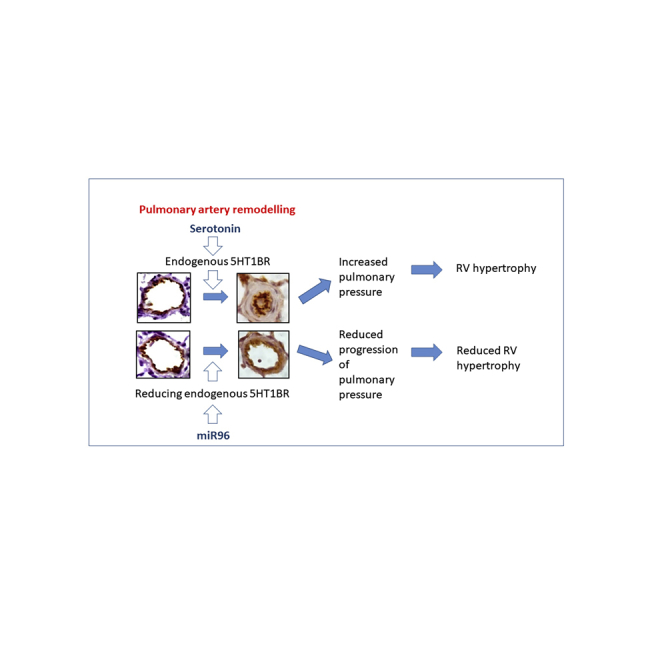

## Teaser

Serotonin, acting through the 5HT1B receptor contributes to the pathophysiology of pulmonary hypertension. In a model of pulmonary hypertension, miR96 delivered directly to the lungs decreased expression of the lung 5HT1B receptor and reversed pulmonary hypertension without causing any inflammation or loss of liver or kidney function.

## Introduction

Pulmonary arterial hypertension (PAH) is a chronic condition defined by vascular remodeling and complex vascular lesion formation arising from accelerated proliferation of pulmonary endothelial, smooth muscle, and fibroblast cells.^1^ Increased activation of the serotonergic system has been implicated in the development and progression of pulmonary hypertension (PH).^2^^,^^3^ This includes increased activation of the serotonin transporter (SERT) and the serotonin 1B receptor (5HT1BR), as well as the synthesis of serotonin via tryptophan hydroxylase 1 (TPH1).^2^^,^^4^ Synthesis of serotonin via TPH1 has been associated with both experimental and clinical PAH,^5^, ^6^, ^7^ and TPH1 inhibitors can reverse experimental PH.^8^ Overexpression of the SERT in female mice leads to increased basal right ventricular (RV) systolic pressure (RVSP) and renders the mice more susceptible to hypoxia-induced PH.^9^ Therefore, inhibition of the serotonin pathway may have beneficial effects in the treatment of PAH.

It is the 5HT1BR that mediates serotonin-induced contraction of conduit and resistance human pulmonary arteries^10^^,^^11^ and serotonin-induced proliferation of female human pulmonary arterial smooth muscle cells (hPASMCs).^12^ 5HT1BR antagonism or knockout can prevent hypoxia-induced PH in rodents.^13^ MicroRNAs (miRNAs) are short, non-coding nucleic acid sequences that can affect downstream systems by directing degradation of mRNA transcripts or silencing mRNA translation. Several miRNAs have subsequently been implicated in the development of PAH,^14^ and many are involved in serotonergic signaling in other systems of the body.^15^^,^^16^ miRNA96 (miR96) is the only known miRNA that targets the 5HT1BR,^12^ and miR96 levels are decreased in female PAH patient hPASMCs when compared to non-PAH controls.^12^ This is accompanied by increased expression of the 5HT1BR. Both increased 5HT1BR expression and proliferation can be inhibited by a miR96 mimic. Hence, decreased miR96 is likely to play a role in the increased serotonin/5HT1BR-induced hyper-proliferation observed in female PAH patient hPASMCs. This suggests that miR96 may offer a novel therapeutic approach to PAH, selectively targeting the serotonin receptor responsible for pathogenic effects. The mechanisms of serotonin/5HT1BR-induced proliferation of hPASMCs are well documented now by ourselves and others,^12^^,^^17^, ^18^, ^19^ so there was no requirement to investigate this further in this study. The novelty of this study is that we deliver the miR96 directly to the lung and evaluate the ability of lung-delivered miR96 to reverse progression of PH in the robust sugen/hypoxia rat model. We also examine the effects of miR96 on liver, kidney, and cardiac function and fibrosis. To compare the effects of the miR96 mimic with those of a known 5HT1BR antagonist, we compared the hemodynamic and off-target effects with those of the 5HT1BR antagonist SB216641. In addition, we determined longer term effects of lung-delivered miR96 and the effects of miR96 on liver and kidney function. This provides novel and unique pre-clinical data to inform potential translational studies.

## Results

### Effects of miR96 on Pulmonary Hemodynamics in the Sugen/Hypoxic Rat

**Figure 1:**
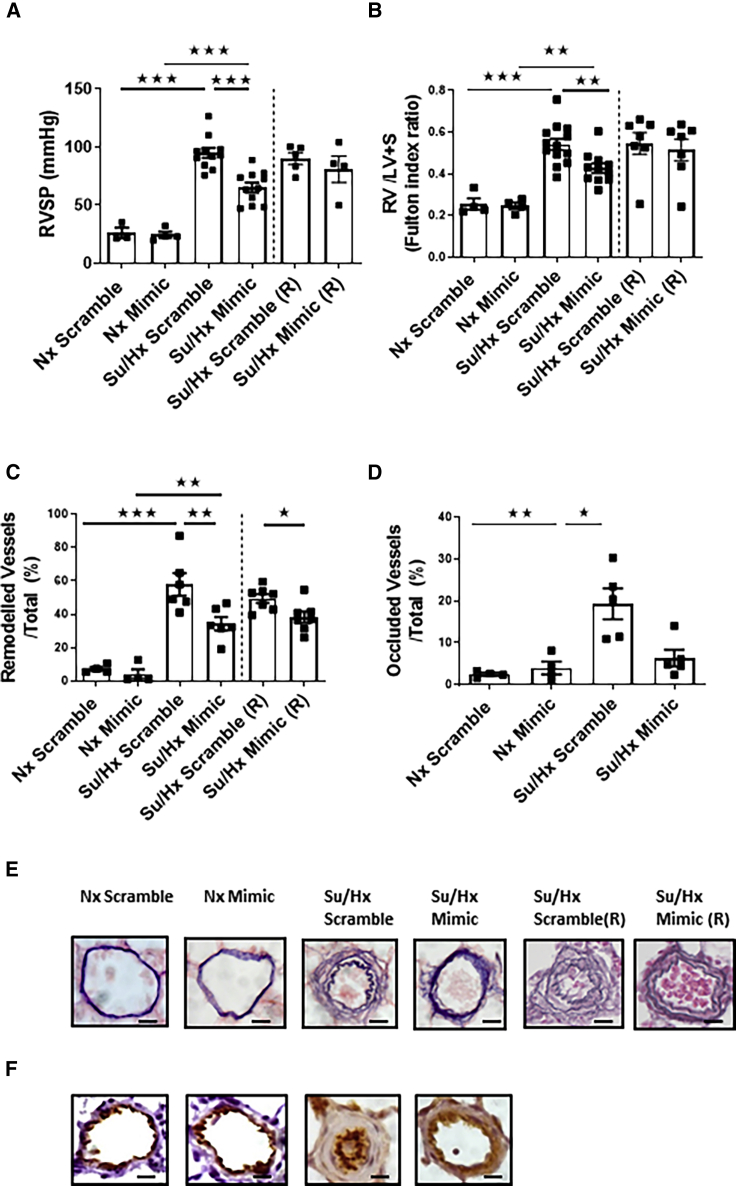
Hemodynamic and Hypertrophic Indicators in the Sugen-Hypoxic Rat Model in the Presence of miR96 (A and B) Right ventricular (RV) systolic pressure (RVSP) (A) and RV hypertrophy, as measured by RV weight/left ventricular (LV) weight + septum weight (B) after normoxia (Nx) or sugen/hypoxia (Su/Hx) with administration of scramble sequence (Scramble) or miR96-mimic (i.t.) (Mimic). n = 4. (C and D) Number of remodeled vessels (C) and occluded vessels (D) after 3 weeks of hypoxia and 3 weeks of Sugen (30 mg/kg) + scramble sequence or miR96-mimic (i.t.) or in Nx conditions. Su/Hx scramble sequence (R) and Su/Hx mimic (R) indicate data (n = 4) taken 3 weeks after the last dose of scramble sequence or mimic. (E and F) Representative images of elastin-picrosirius-red-stained (E) and Von-Willebrand-stained (F) distal pulmonary artery endothelial cells. Error bars indicate mean ± SEM. n = 3–4 for Nx, and n = 6–13 for Hx. Statistical significance was determined by one-way ANOVA with Tukey’s post hoc test. ^★^p < 0.05; ^★★^p < 0.01; ^★★★^p < 0.001. Scale bars, 20 μm.

RVSP was increased in the sugen/hypoxic rats, and miR96 reduced RVSP by ∼30% (Figure 1A). RV hypertrophy (RVH) was doubled in the sugen/hypoxic rats, and miR96 delivery reduced RVH by ∼22% (Figure 1B). The percentage of remodeled pulmonary arteries was markedly increased in the sugen/hypoxic scramble sequence control group versus the normoxic control group, and miR96 reduced this by ∼40% (Figure 1C). The number of occluded vessels was not significantly increased in the miR96 treated animals (Figure 1D). Representative images of remodeled arteries are shown in Figure 1E, and images of occluded arteries stained for endothelial cells are shown in Figure 1F. Three weeks after the final dose of miR96, the hemodynamic and histological analyses were repeated in a further group of rats to investigate whether there was any recovery or longer term effects of miR96. RVSP had recovered to pre-miR96 levels, as had RVH, and there was no further effect of miR96 (Figures 1A and 1B). The percentage of remodeled vessels had recovered slightly, but the inhibitory effect of miR96 remained after 3 weeks (Figure 1C).

### Effects of a 5HT1BR Antagonist on Pulmonary Hemodynamics in the Sugen-Hypoxic Rat

**Figure 2:**
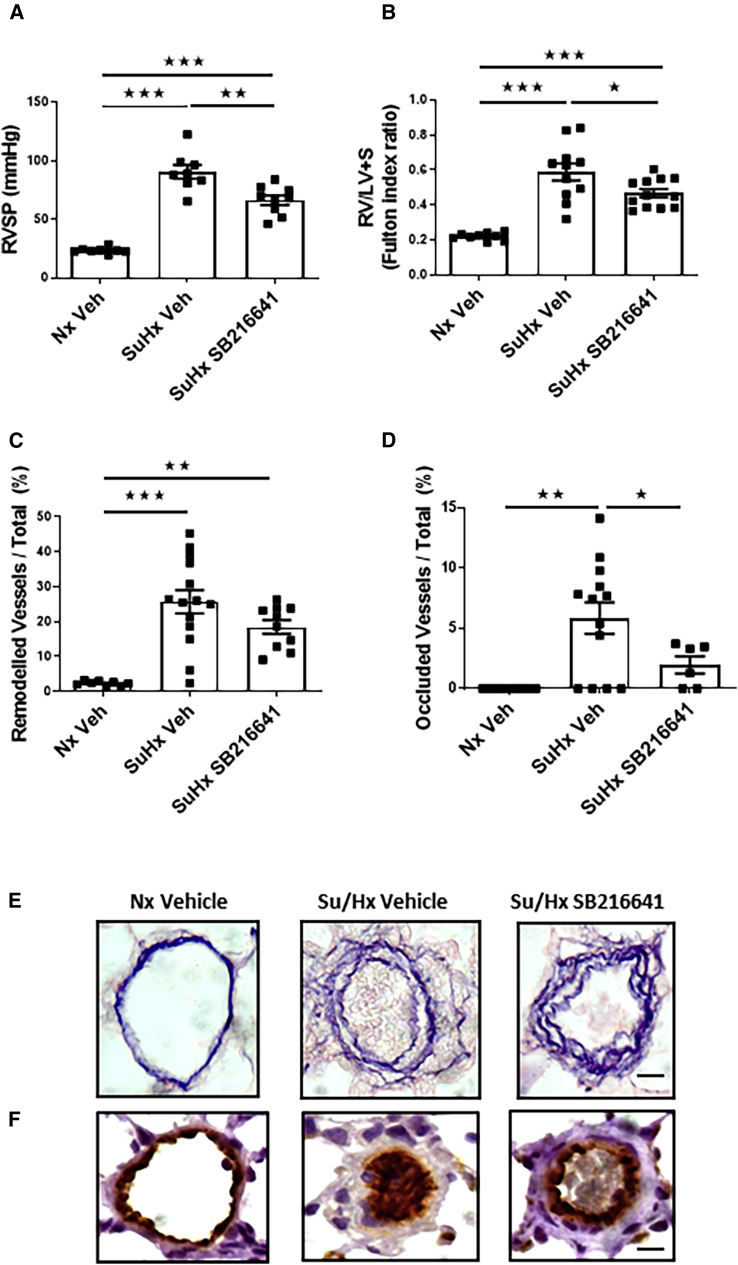
Hemodynamic and Hypertrophic Indicators in the Sugen-Hypoxic Rat Model in the Presence of the 5HT1BR Antagonist SB216641 (A and B) RVSP (A) and RV hypertrophy, as measured by RV weight/LV weight + septum weight (RV/LV+S) (B) in Nx vehicle (Veh)-treated group compared to Su/Hx Veh-treated and sugen-hypoxic antagonist-treated group. (C and D) Number of remodeled vessels (C) and occluded vessels (D) in Nx-Veh-treated group compared to Su/Hx Veh-treated and Su/Hx antagonist-treated group. (E and F) Representative images of elastin-picrosirius red-stained distal pulmonary arteries (E) and Von Willebrand-stained distal pulmonary arteries (F). Error bars indicate mean ± SEM; n = 4–11 for each group. Statistical significance was determined by one-way ANOVA with Tukey’s post hoc test. ^★^p < 0.05; ^★★^p < 0.01; ^★★★^p < 0.001. Scale bars, 20 μm.

SB216641 reduced RVSP and RVH by ∼38% and ∼20%, respectively, in the sugen/hypoxic rats (Figures 2A and 2B). SB216641 had no significant effect on the total percentage of remodeled vessels (Figure 2C) but reduced the percentage of occluded lesions in the sugen/hypoxic rats (Figure 2D). Representative images of remodeled arteries are shown in Figure 2E, and images of occluded arteries stained for endothelial cells are shown in Figure 2F.

### Effects of miR96 and 5HT1BR Antagonism on Markers of Cardiac Hypertrophy and Fibrosis

**Figure 3:**
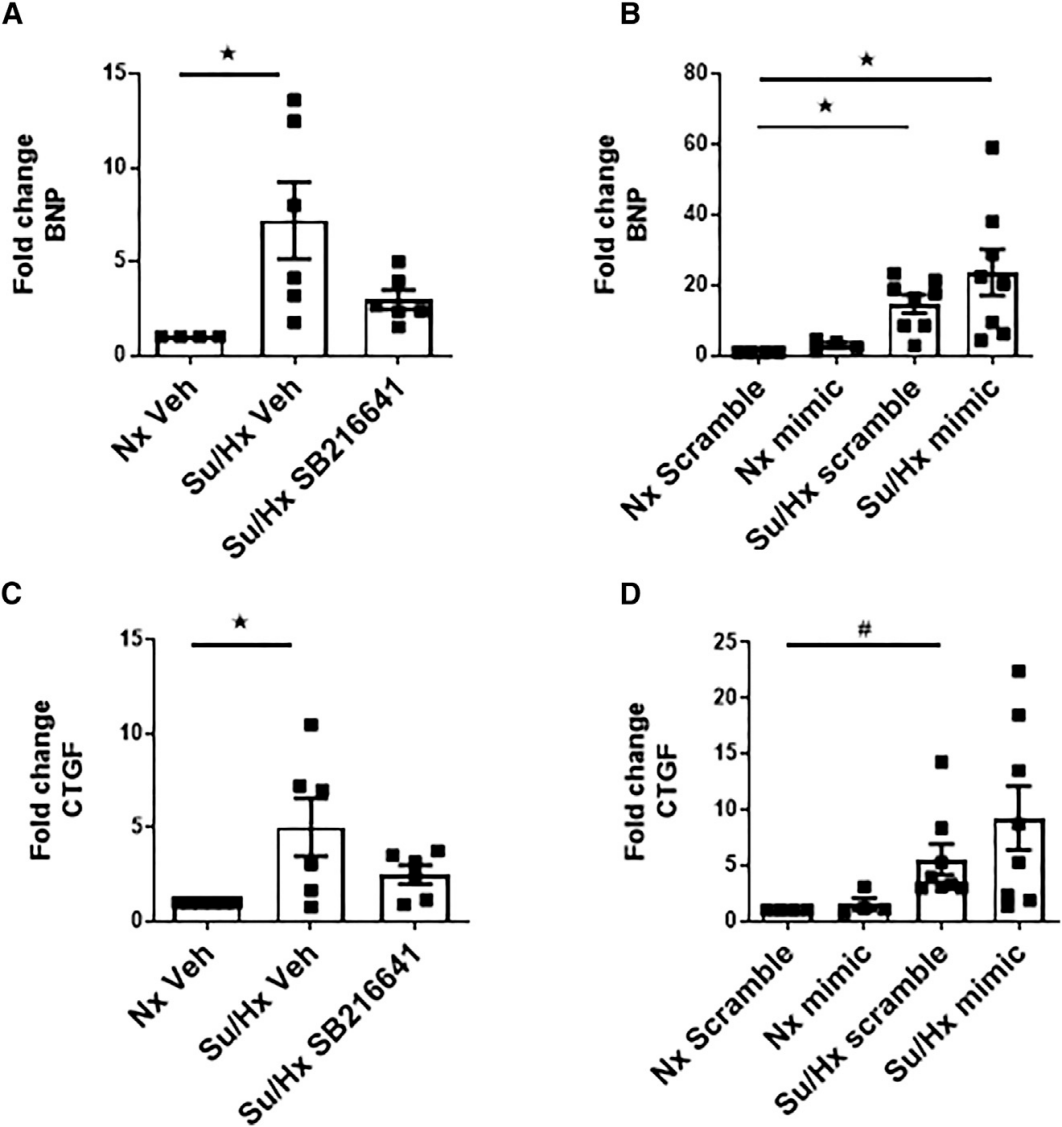
Effects of SB216641 and miR96 (Mimic) on mRNA Transcript Expression of Hypertrophic and Fibrotic Markers in the Right Ventricle: Expression in RV Tissue from Nx Veh and Su/Hx Rats (A–D) BNP (*NPPB*): SB216641 (A); BNP: mimic (B); CTGF: SB216641 (C); and CTGF: mimic (D). Error bars indicate mean ± SEM. A one-way ANOVA with Tukey post hoc test was used. ^★^p < 0.05; ^#^p < 0.05, Student’s unpaired t test. n = 4–8.

B-type natriuretic peptide (*BNP*; *NPPB*) gene expression was increased in the sugen/hypoxic right ventricle (Figures 3A and 3B). After SB216641 administration, *BNP* mRNA levels were no longer significantly higher than in the vehicle controls (Figure 3A). BNP levels were not affected by miR96 (Figure 3B). RV mRNA levels of connective tissue growth factor (*CTGF*) were significantly increased in the sugen/hypoxic rats (Figures 3C and 3D), and mRNA levels were normalized by the 5HT1BR antagonist (Figure 3C) but were unaffected by miR96 (Figure 3D).

Neither miR96 nor SB216641 affected RV expression of collagen 1A1 (COL1A1), transforming growth factor-β (*TGFβ*), GATA binding protein 4 (*GATA4*), or myosin heavy chain-β/α (*MHCβ/MHCα*) (Figures S1A–S1H). *BNP* or *MHCβ/MHCα* mRNA ratios (Figures S2A–S2D) and *COL1A1* and *CTGF* expression (Figures S3A–S3D) were unaffected in the sugen/hypoxic rat in the left ventricle.

### Effects of miR96 and a 5HT1BR Antagonist on RV Function

**Figure 4:**
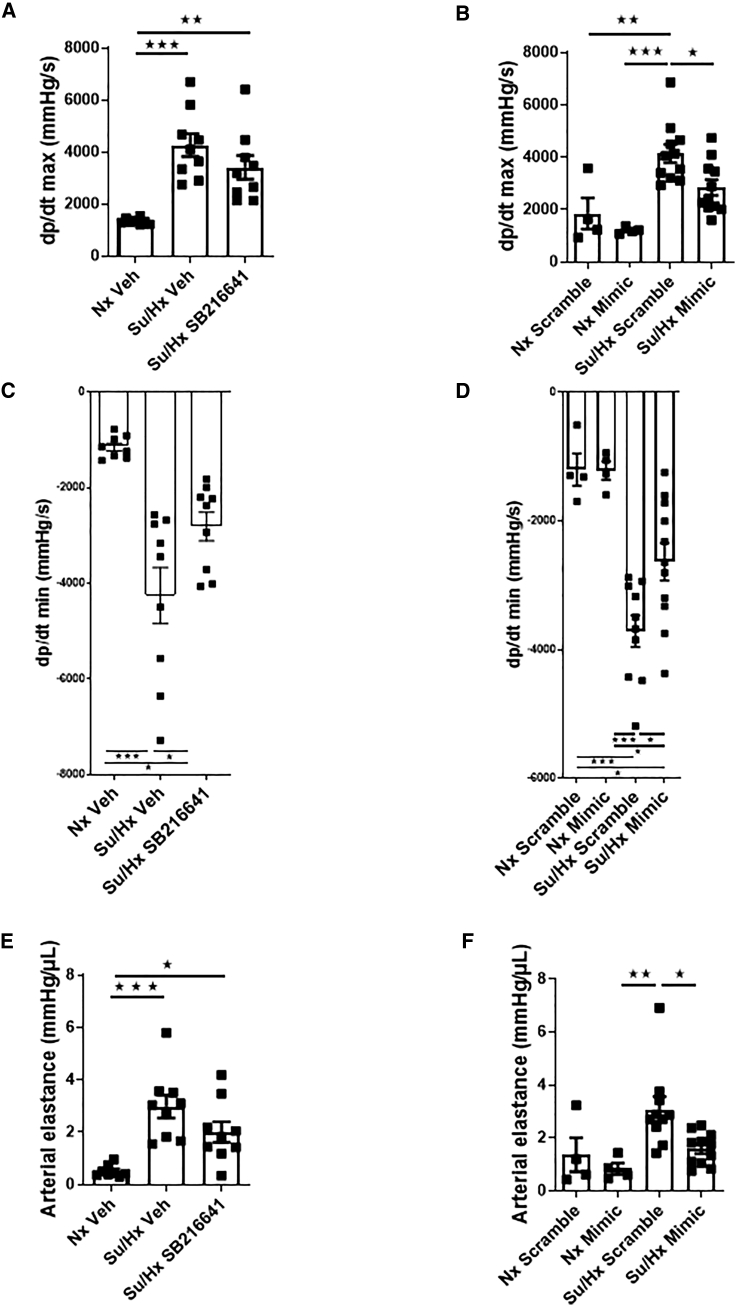
Hemodynamic Assessment of RV Function Su/Hx Rat Model in the Presence of miR96 Mimic or SB216641 (A–F) RV inotropy as measured by dp/dt max (A and B), lusitropy as measured by dp/dt min (C and D), and RV load as measured by arterial elastance (E and F). Nx Veh, normoxic vehicle; Su/Hx Veh, sugen/hypoxic vehicle; Su/Hx SB216641, sugen/hypoxic group treated with SB216641; Nx scramble, normoxic scramble-sequence-treated group; Nx Mimic, normoxic group treated with miR96 mimic; Su/Hx Scramble: sugen/hypoxic group treated with scramble sequence; Su/Hx Mimic: sugen/hypoxic group treated with miR96 mimic. Error bars indicate mean ± SEM. One-way ANOVA with post hoc Tukey test was used to assess statistical significance. ^★^p < 0.05; ^★★^p < 0.01; ^★★★^p < 0.001. n = 4–8.

RV contractility was measured by the maximum derivative of pressure/derivative of time (dP/dt max), which was increased in the sugen/hypoxic rats; however, this was not affected by SB216641 (Figure 4A) but was reduced by miR96 (Figure 4B). Ventricular lusitropy (minimum derivative of pressure/derivative of time; dP/dt min) was reduced by SB216641 (Figure 4C) and by miR96 (Figure 4D). RV load was increased in the sugen/hypoxic rats (Figure 4E). This was not affected by SB216641 (Figure 4E), but progression was reduced by miR96 (Figure 4F).

**Figure 5:**
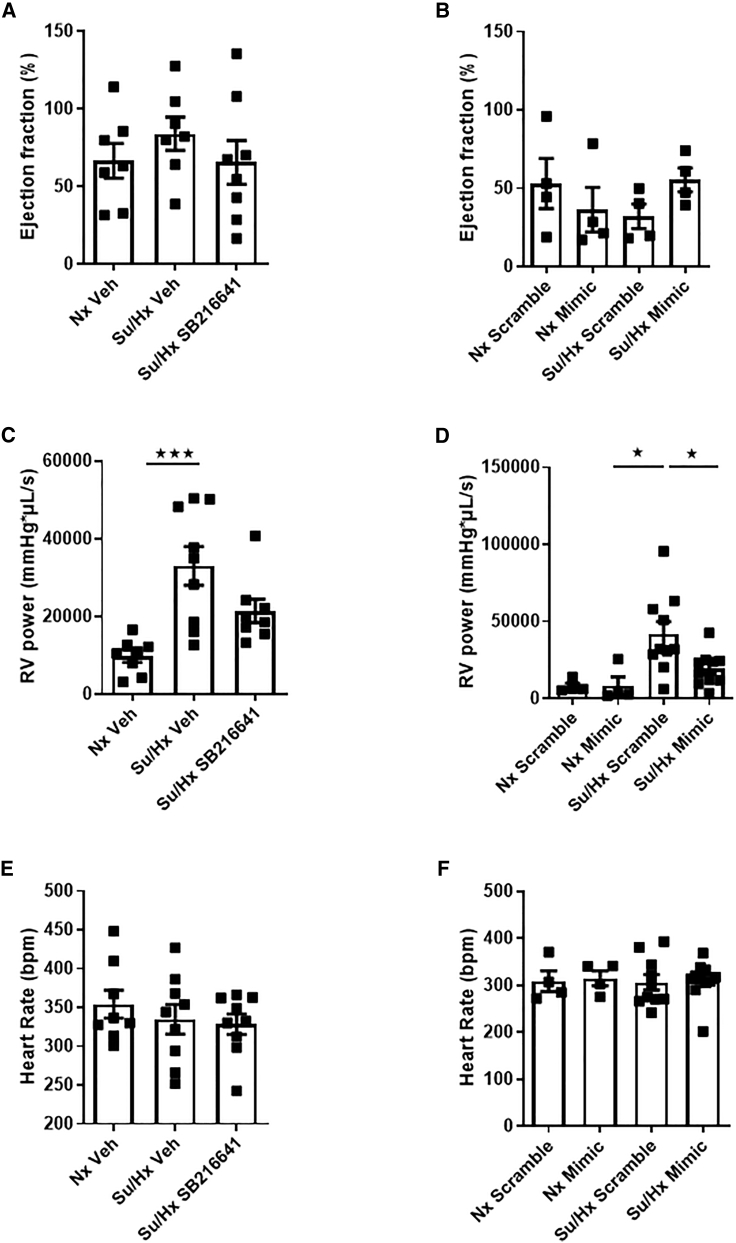
Hemodynamic Assessment of RV Function Su/Hx Rat Model in the Presence of miR96 Mimic or SB216641 (A–F) Ejection fraction (A and B), RV loop power (C and D), and heart rate (E and F) in Nx Veh and Su/Hx and SB216641/Veh-treated rats or in normoxic scramble and normoxic miR96 mimic-treated versus Su/Hx scramble sequence-treated and Su/Hx miR96-mimic-treated rats. Error bars indicate mean ± SEM. One-way ANOVA with post hoc Tukey test was used to assess statistical significance. ^★^p < 0.05; ^★★★^p < 0.001. n = 4–8.

Ejection fraction was unaffected by miR96 or SB216641 (Figures 5A and 5B). RV loop power was increased in the sugen/hypoxic rat (Figures 5C and 5D), and this progression was prevented by miR96 (Figure 5D). Heart rate was unaffected by miR96 or SB216641 (Figures 5E and 5F).

### Effects of miR96 on 5HT1BR Expression in the Lung

**Figure 6:**
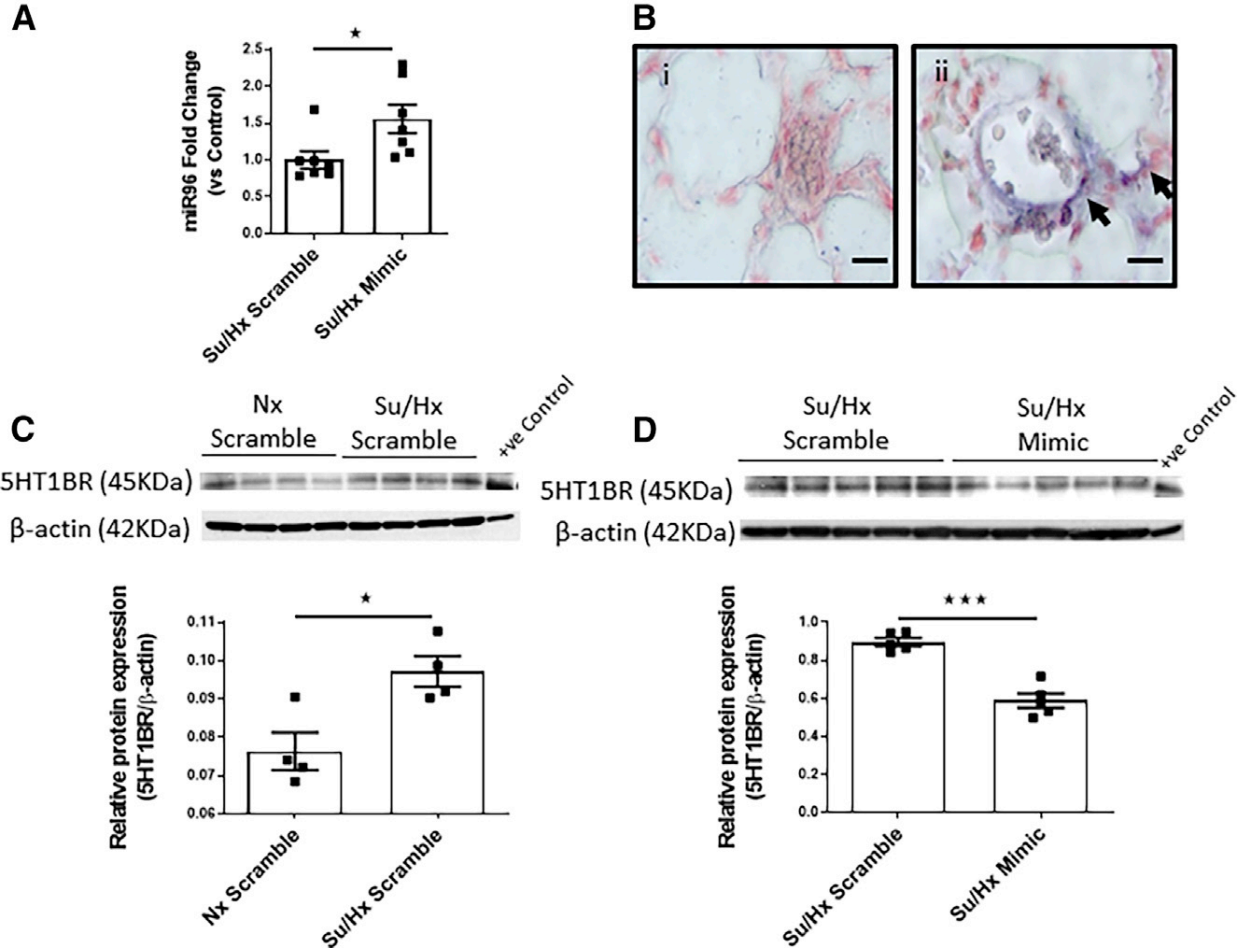
Expression of miR96 Mimic in the Lung and Pulmonary Artery (A) miR96 expression in the lungs of Su/Hx scramble sequence and Su/Hx miR96 mimic-treated rats. (B) miR96 expression in the pulmonary artery of Su/Hx scramble sequence (i) and Su/Hx miR96-mimic-treated rats (ii); miR96 (purple) is indicated by arrows. (C and D) 5HT1BR protein expression in whole lung tissue from normoxic scramble sequence (C) and Su/Hx miR96 mimic-treated rats (D). Data are expressed as the ratio of expression (target/loading control). Error bars indicate mean ± SEM. One-way ANOVA with post hoc Tukey test was used to assess statistical significance. ^★^p < 0.05; ^★★★^p < 0.001. Scale bars, 20 μm.

miR96 mRNA expression was increased by ∼33% in the lungs after administration of miR96 (Figure 6A). *In situ* analysis of miR96 expression demonstrated that miR96 uptake was not uniform across the lung but appeared in discrete regions. Figure 6B demonstrates that miR96 expression was localized to the vascular wall of small pulmonary arteries, and this was not observed after administration of the scramble sequence. There was an increase in 5HT1BR expression within the sugen/hypoxic rat lungs (Figure 6C), and miR96 decreased 5HT1BR expression in the sugen/hypoxic rat lung (Figure 6D).

BMPR2 protein expression was not altered in the sugen/hypoxic scramble sequence group, compared to the normoxic controls (Figure S4A), but was increased by miR96 in the sugen/hypoxic rat lung (Figure S4B). Three weeks after the last dose of miR96, increased miR96 expression was still measurable in the lungs of the sugen/hypoxic rats (Figure S4C), although the increase was only ∼21% compared to ∼33% 3 weeks before (Figure 6A).

### Effects of miR96 on Liver and Kidney Function and Inflammatory Responses in the Lung

We investigated possible renal and hepatic toxicity in the miR96-treated animals. An increase in plasma alanine aminotransferase (ALT) would indicate liver damage or inflammation and elevated creatinine levels signifiy impaired kidney function. miR96 did not have any adverse effects on rat plasma ALT levels (Figure S5A) or on creatinine levels in rat plasma and rat urine (Figure S5B). miR96 also had no effects on fibrotic mRNA markers *COL1A1*, *COL3A1*, and fibronectin (*FN1*) in the liver (Figures S5C–S5E). Similarly, miR96 had no effect on the gene expression of the inflammatory markers interleukin-6 (*IL6*) and tumor necrosis factor α (*TNFα*) or the chemokine ligand 5 (*CCL5*) in the lung (Figures S6A–S6C). There was also no effect of miR96 on mast cell accumulation in the lung (Figures S6D and S6E).

### miR96 Expression in Other Tissues, Large Pulmonary Arteries, and Airways

Lung delivery of miR96 did not affect miR96 expression in liver (Figure S7A), kidney (Figure S7B), or RV tissue (Figure S7C). *In situ* localization also shows that miR96 expression was present in the smooth muscle and endothelium of large proximal pulmonary arteries and the smooth muscle of airways of miR96-treated rats (Figures S8A–S8C).

## Discussion

This is the first study to demonstrate that miR96 can be delivered directly to the lungs in a model of occlusive PH with increased expression in pulmonary arteries inducing therapeutic effects. This is associated with miR96 targeting 5HT1BR and decreasing lung expression, as we demonstrated previously.^12^ We have determined secondary therapeutic effects of lung-delivered miR96 on RV function and shown that transfection was not associated with increases in inflammatory markers or lung fibrosis and that liver and kidney function was not affected. In addition, we demonstrate therapeutic effects of a systemically delivered 5HT1BR antagonist in this model for the first time.

We assessed the effectiveness of direct delivery of miR96 to the lungs in the sugen/hypoxic rat model of PH. In this model, after initial hypoxic exposure, the PH increases with time once the animals are exposed to normoxic conditions.^20^ The model demonstrated elevated RVSPs (over 90 mmHg), profound RV hypertrophy, and fibrosis, as well as pulmonary vascular remodeling and occlusive lesions in the distal pulmonary arteries. RV contractility was elevated in the rat model, suggesting that this is a model of early-stage PH where the right ventricle has adapted to afterload with increased contractility. Consistent with being a model of PH, the cardiac effects were only observed in the right ventricle, with no changes in the left ventricle. It should be noted, however, that one limitation of this study is that hemodynamic parameters, including heart rate, are affected by anesthesia.

After direct delivery of miR96 to the lung, as expected, we observed increased expression of miR96 in the large proximal pulmonary arteries and airways but also in the smooth muscle of small distal pulmonary arteries. The distribution of miR96 appeared in discrete areas of the lung, as it is distributed in one inhalation. This is similar to the situation for inhalation of bronchodilators and anti-inflammatory agents, where it has been shown that only 20% of drug is delivered, with the majority being swallowed.^21^ The observation that, despite this, miR96 was effective suggests that this approach has much potential for development. Direct delivery of miR96 to the sugen/hypoxic rat lung also decreased 5HT1BR expression in small pulmonary arteries. This is consistent with our previous studies showing miR96 downregulates the 5HT1BR.^12^ To date, miR96 is the only miRNA that has been identified as targeting *5HT1BR*. miR96 reduced the progression of pulmonary vascular remodeling and inhibited the development of occlusive pulmonary lesions. miR96 also reduced the progression of increases in RVSP and RVH. As we could not detect increased miR96 expression in RV tissue after i.t. administration, these therapeutic effects are secondary to improvements in the pulmonary circulation. BNP is released in response to changes in pressure inside the heart and is a marker of cardiac dysfunction.^22^ While *BNP* gene expression was elevated in the right ventricle of the sugen/hypoxic rats, miR96 delivered to the lungs did not affect BNP gene expression in the right ventricle. Likewise, miR96 did not normalize elevated *CTGF* expression, a marker of cardiac fibrosis. This reflects the administration of miR96 directly to the lungs; however, it is possible that these adaptive changes might have resolved with longer term treatment with miR96, secondary to resolution of vascular changes in the pulmonary circulation.

We wanted to determine the longer term effects of miR96 and so studied effects on the pulmonary circulation 3 weeks after miR96 administration. While there was still an increase in lung miR96 expression, this was reduced by 12%, compared with expression 3 weeks earlier. After 3 weeks, miR96 had no effects on RVSP, RVH, or pulmonary occlusions, but the inhibitory effects on pulmonary vascular remodeling were still evident. This is consistent with the vascular remodeling being the last index of PH to resolve in the hypoxic model of PH when rats are removed from hypoxia and placed in normoxia.^23^ This would indicate that weekly maintenance doses of miR96 might be required therapeutically.

Right heart failure can arise from both contraction (inotropic) and relaxation (lusitropic) abnormalities. Previous studies have shown that adaptive responses in these indices are associated with PAH.^24^ The therapeutic effects of miR96 on the pulmonary circulation caused a secondary reversal of RV contractility and RV load. The increased contractility is an adaptive response of the right ventricle, and reversal is consistent with a decrease in afterload. Consistent with this, RV load, or pulmonary effective arterial elastance (Ea) (measure of total RV afterload) was also reduced by miR96.

The orally administered 5HT1BR antagonist was also effective in reducing RVSP and RVH. Due to compound precipitation issues, this compound could not be delivered directly to the lungs. SB216641 reduced the pulmonary occlusive lesions in the rat model. This is consistent with previous studies where we demonstrated that a 5HT1BR antagonist and *HTR1B* knockdown protected mice from hypoxia-induced PH.^13^ However, at this dose, SB216641 did not significantly reduce the percentage of remodeled pulmonary arteries in the sugen/hypoxic rat where ∼60% of vessels were remodeled. An alternative 5HT1BR antagonist did reverse remodeling in the hypoxic rat, where this was less severe (25%).^13^ In this regard, therefore, miR96 was more effective than the 5HT1BR antagonist. This could have been due to increased reduction in receptor expression being more effective than competitive antagonism or because miR96 was having the additional therapeutic effects observed and discussed earlier. 5HT1BRs are Gi-coupled and are subject to significant pharmacological synergy,^25^, ^26^, ^27^, ^28^ which may overcome 5HT antagonism to a greater extent than a reduction in receptor number. The 5HT1BR antagonist also normalized *BNP* mRNA levels in the right ventricle, consistent with its having been administered orally. SB216641 reduced the adaptive increase in lusitropy (dP/dt min) but did not affect inotropy or RV load. This likely reflects its more moderate effects (versus miR96) in reversing RVSP and RV hypertrophy, as well as the lack of effects on the percentage of remodeled pulmonary arteries (hence, less reduction in afterload).

We previously demonstrated that female (not male) PAH patient hPASMCs overexpress the 5HT1BR and exhibit increased serotonin-induced proliferation, mediated by the 5HT1BR. Accordingly, transfection of these cells with miR96 both reduced the expression of the 5HT1BR and inhibited serotonin-induced proliferation.^12^ The increased expression of the 5HT1BR in female PAH hPASMCs is likely to be due to the effects of estrogen, which can induce expression of the 5HT1BR in these cells.^29^

We and others have previously determined molecular mechanisms of 5HT1BR stimulation in the pulmonary circulation and in PASMCs, so there was no requirement to investigate this further in the present study. Through the 5HT1BR, serotonin induces Src-related, kinase-regulated, Nox1-induced reactive oxygen species (ROS) and Nrf-2 dysregulation. This contributes to increased post-translational oxidative modification of proteins and activation of redox-sensitive signaling pathways in hPASMCs and is associated with the mitogenic responses in hPASMCs.^19^ Rho A/Rho kinase also participate in 5HT1BR-mediated mitogenesis through an effect on cytoplasmic-to-nuclear translocation of ERK1/ERK2.^30^ Serotonin can transactivate the serine kinase receptor BMPR1A to activate Smads 1/5/8 via Rho and Rho kinase transglutaminase. This also contributes to serotonin-induced PASMC proliferation via AKT signaling.^31^ In addition, we have shown that 5HT1BR activity is enhanced by increased vascular tone through pharmacological synergy.^28^

miR96 increased lung BMPR2 protein expression in the sugen/hypoxic rats. BMPR2 expression and activity are crucial in the pulmonary vasculature and mutations in *BMPR2* underlie the vast majority of heritable forms of PAH.^32^^,^^33^ Furthermore, many idiopathic cases of PAH are a result of dysfunctional BMPR2 signaling.^34^ Therefore, increased BMPR2 signaling may have contributed to the therapeutic effects of miR96. We have also previously shown that there is increased 5HT1BR gene and protein in *BMPR2* mutation knockin mice, suggesting an inverse relationship between 5HT1BR expression and BMPR2 signaling.^12^

It was important to investigate any off-target effects of i.t.-administered miR96. miR96 had no effects on liver or kidney function. Mast cells are markers for allergic and inflammatory responses.^35^ The absence of inflammatory mRNA markers or mast cell numbers in the lungs after miR96 indicates that miR96 itself does not cause any off-target inflammation that may be detrimental to the health of the animal. This suggests that direct delivery of the miR96 mimic to the lungs may prevent excessive off-target effects that may be produced by intravenous delivery or other approaches.

Other limitations of this study include the necessity to study open-chested anesthetized animals and financial constraints limiting our ability to study the effects of dosing with miR96 over a longer period of time. Our whole-lung expression approach, required to harvest sufficient tissue, did not allow us to identify potential cellular compartmentalization of the miR96.

In conclusion, our studies have shown that, in the sugen/hypoxic rat model, miR96 delivered directly to the lungs is therapeutic and tolerable, with no observed side effects in the kidney, in the liver, or on inflammatory markers. We demonstrate that miR96 may exert therapeutic effects through decreased 5HT1BR and signaling, leading to decreased progression of pulmonary vascular remodeling.

## Materials and Methods

### In Vivo

The procedures conform to the guidelines from Directive 2010/63/EU of the European Parliament on the protection of animals used for scientific purposes. All animal procedures performed were also approved by the UK Home Office according to regulations regarding experiments with animals in the United Kingdom (ASPA 1986) and therefore approved by local ethical review (#P15C58D3F).

Female Sprague-Dawley rats (Charles River Laboratories), 125–150 g, were dosed subcutaneously with 30 mg/kg sugen and exposed to 21 days of hypobaric hypoxia (550 mbar) modified from methods described previously.^36^^,^^37^ Rats were then maintained in normoxia for 21 days. Control rats were maintained in normoxic conditions. Treatment with drug or miR96 started immediately after the hypoxic/control period (day 21). Rats were treated for 3 weeks as follows: (1) ddH_2_O/vehicle or 5HT1BR antagonist SB216641 (dosed orally, daily [7.5 mg/kg]); (2) negative scramble sequence control or miR96 mimic (Applied Biosystems) delivered with Invivofectamine (Thermo Fisher Scientific, Winsford, UK) prepared in accordance with the manufacturer’s instructions (dosed weekly intra-tracheally [100 μL; 50 μg per rat per week for 3 weeks]). Rat cardiac hemodynamics were measured using the Millar Pressure-Volume (PV) Loop System after the 3 weeks of interventions.^37^ Anesthesia was delivered via a facemask using 3% isofluorane with an air/oxygen mixture at a flow rate of 1 L/min. Depth of anesthesia was continuously monitored throughout to ensure that the animal remained unconscious and insentient. This was assessed by pedal withdrawal reflex and visual monitoring of respiration rate and rhythm. Euthanasia was carried out under terminal anesthesia by exsanguination via cardiac puncture before heart and lungs were removed.

An additional group of rats were left for 3 weeks after the last dose of miR96 or scramble sequence control before their hemodynamics were measured. This was in order to test the longer term effects of miR96. See the Supplemental Information for further details.

### RVH

RVH was assessed as the right ventricle weight expressed as a ratio to the left ventricle plus septum weight (Fulton index ratio).^37^ See the Supplemental Information for further details.

### qRT-PCR

Briefly, all RNA was extracted using the miRNeasy Mini Kit following the manufacturer’s protocol (QIAGEN, Manchester, UK). RNA was then reverse transcribed to cDNA before being probed with specific TaqMan primer-probe sets (Thermo Fisher Scientific, Winsford, UK).^38^ See the Supplemental Information for further details.

### Western Blotting

Protein expression was assessed by immunoblotting in whole lung tissue.^22^ See the Supplemental Information for further details.

### Creatinine Assay

Plasma and urine creatinine levels were measured using the 96-well colorimetric Creatinine Assay Kit (ab65340, Abcam, Cambridge, UK) following the manufacturer’s protocol.

### ALT Assay

Rat plasma ALT levels were assessed using the colorimetric 96-well Rat ALT ELISA Kit (ab234579, Abcam, Cambridge, UK).

### Histology

Pulmonary artery remodeling and lung mast cell staining were assessed using histological techniques that have been described previously.^39^ See the Supplemental Information for further details.

### *In Situ* Hybridization

Localization of miR96 within the lung was assessed using *in situ* hybridization as described previously^12^^,^^40^ and in the Supplemental Information.

### Statistical Analysis

Either a one-way ANOVA with Tukey’s post hoc test was used to determine significance of variances (as indicated in the figure legend of each figure), or unpaired Student’s t test was used where appropriate. All graphs and statistical analyses were produced and performed using GraphPad Prism v.5. All data are presented as mean ± SEM, and p <0.05 was considered statistically significant.

## Author Contributions

Conceptualization, M.R.M. and C.K.D.; Investigation, C.K.D., S.F., D.H., M.N., R.L.O., and H.L.; Formal Analysis, C.K.D., M.N., H.L., R.L.O., and N.D.; Writing and Formatting, C.K.D., N.D., and M.R.M.; Funding Acquisition, M.R.M.

## Conflicts of Interest

The authors declare no competing interests.

## Supplemental Information

Document S1. Supplemental Materials and Methods, Tables S1 and S2, and Figures S1–S9

Document S2. Article plus Supplemental Information
