# Supplementary material for: Direct Delivery of MicroRNA96 to the Lungs Reduces Progression of Sugen/Hypoxia-Induced Pulmonary Hypertension in the Rat
Source: Mol Ther Nucleic Acids. 2020 Sep 6;22:396–405. doi: 10.1016/j.omtn.2020.09.002 (PMC7533346; doi:10.1016/j.omtn.2020.09.002)
Supplement: Document S1. Supplemental Materials and Methods, Tables S1 and S2, and Figures S1–S9 [file mmc1.pdf]

OMTN, Volume *22*

Supplemental Information

Direct Delivery of MicroRNA96 to the Lungs Reduces Progression of Sugen/Hypoxia-Induced Pulmonary Hypertension in the Rat

Craig K. Docherty, Nina Denver, Simon Fisher, Margaret Nilsen, Dianne Hillyard, Rebecca L. Openshaw, Hicham Labazi, and Margaret R. MacLean

Online Data Supplement

Materials and methods

In vivo

A microsprayer 1A-1B (Penn Century) and gas-tight SGE syringe (Hamilton syringe) was primed with PBS prior to procedure and then sterilized with ethanol. Female Sprague Dawley rats (Charles River), 125-150g, were induced into deep anesthesia using using 5% Isofluorane with an air/oxygen mixture at a flow rate of 1L/min into an induction chamber for 5 to 7 minutes to ensure deep anaesthesia. Animals are assessed by pedal withdrawal reflex and monitoring of respiration rate and rhythm. After suspending by incisors at a 45 degree angle, blunt forceps were used to position the tongue so it did not obscure the trachea. The microsprayer was loaded with 100µL of miR96 mimic or scramble sequence and slowly inserted past the epiglottal space and vocal cords to the region directly above the bifurcation of the bronchi, visualized using a light source. The sprayer plunger was quickly and forcefully depressed and held in place for 10 seconds. Animals were then held upright for a further 10-15 seconds before recovery in a warm cage. Animals were closely observed until fully recovered and placed back into their original cage.

Hemodynamic measurements

Rats were anesthetized in 3-4% (v/v) isoflurane, the level of anesthesia was assessed by absence of pedal reflex to toe pinch. The rats were placed on a heating pad, where the rat's front paws and one distal paw were taped down. Following a neck midline incision, the pretracheal muscles were pulled apart gently with forceps and dissected the around the trachea. A surgical silk suture was placed underneath the trachea, a small cut onto the surface of the trachea was made into which the tracheotomy cannula was inserted and secured with the silk suture. Then, the rats were immediately transferred to a ventilator where they were maintained at approximately 1.5-2% (v/v)

isoflurane supplemented with a constant flow of medical oxygen (0.5L/min). Body temperature (37 °C) was monitored by a probe that was inserted into the rat's rectum. Prior to performing open chest surgery, the level of anesthesia of was assessed by absence reflex to toe pinch. Respiratory rate and tidal volume were adjusted for each individual rat based on its weight. Following an incision made over the xyphoid process, a cut was made through the chest wall moving laterally on both sides until the diaphragm is clearly visible, then a thoracotomy was performed to expose the chest cavity. Following a gentle removal of the pericardium from the heart with forceps, a needle was used make a hole into the right ventricle, through which the SPR-869 catheter (A Miller (UK) SPR-869 catheter, with an effective length of 12.5 cm and 4 platinum electrodes, and a pressure sensor centred between pairs of electrodes, was used as per the manufacturer's instructions) was inserted into the right ventricle allowing right ventricular systolic pressure (RVSP) measurements which were recorded using PowerLab data acquisition system with its corresponding software (LabChart Pro). Other cardiac parameters were also measured using the pressure-volume (PV) loop system which were calculated automatically using the Labchart 8 PV loop module software; maximum derivative of pressure/ derivative of time (dp/dt max) which is an indirect measure of cardiac contractility (inotropy), minimum derivative of pressure/ derivative of time (dp/dt min) is an indirect measure of cardiac relaxation (lusitropy), arterial elastance (RV pressure at end of systole/ (max volume - min volume)) is a measure of RV load, ejection fraction which is the volumetric fraction of fluid ejected during each heartbeat, stroke volume which is the maximum volume minus the minimum volume during a PV loop/cardiac cycle, stroke work (area enclosed by the PV loop) and RV power is a measure of ventricular efficiency/workload using maximum values of pressure and volume. All analysis was carried out blind and by licensed, experienced researchers. For vena cava occlusions (to examone cardiac response over a range of preloads and can be obtained with IVC constriction?. a suture was placed beneath the inferior vena cava (VC), at the end of the normal PV

loop recording, using a needle clamp the suture was gently and slowly raised over 1-2 sec.

Histopathology

Pulmonary vascular remodelling was assessed using 5μm sections cut and stained (4-6 sections per lung) using Millers elastin/Picro Sirius Red for identification of vascular remodelling, characterized by a distinctive double elastic lamina. The total number of remodelled vessels (≥80 per lung) was expressed over the total number of vessels present in a lung section in a blinded fashion. Occluded vessels/lesions were counted using lung sections stained with Von Willebrand staining. Mast cells were identified in lung sections stained with toluidine blue and counted per lung section. Images were acquired using a Zeiss Axiocam 305 color (Carl Zeiss Microscopy GmbH, Germany).

MiR96 in-situ hybridization

5μm lung tissue sections were deparaffinised in xylene, rehydrated by passing through graded ethanol (100%, 90%, 70%) then washed for 5 minutes in sterile PBS. Sections were treated with 400μg/ml proteinase K (Invitrogen, 25530049) at 37°C for 10 minutes followed by 2 x 5-minute washes in sterile PBS and dehydrated: 2 x 1 minute washes in 70%, 90% and 100% ethanol. Sections were then air dried for 15 minutes. DIG labelled probes complementary to miR96 (Qiagen) and U6 (Exiqon) were diluted in hybridisation buffer (Sigma, H7782) and hybridised at 52°C for 1-2 hours in a humidified chamber. This was followed by stringency washes of 5 x saline – sodium citrate buffer (SSC) at room temperature, 5 x SSC, 1 x SSC and 2 x SSC at 52°C, then 2 x SSC at room temperature. Sections were then washed in PBS-T before blocking (blocking reagent, Roche 11175041910, in maleic acid buffer) for 15 minutes at room

temperature and sections were incubated with 1:500 alkaline phosphatase AP conjugated antibody for DIG (Roche, 11175041910) in blocking solution. Sections were washed 2 x 3 minutes in PBS-T then incubated at room temperature in NBT/BCIP colour reagent (Roche, 11175041910) in substrate solution (0.1M Tris-HCl, 0.1M NaCl, pH9.5) for 6 days, protected from light. Sections were washed 5 minutes in 0.1M Tris-HCl, 2 x 1-minute water, counterstained with nuclear fast red (1 minute at room temperature), rinsed in tap water for 10 minutes then dehydrated as before and mounted using permount mounting media (Fisher, 15820100). All reagents were made using RNAse-free/ DEPC water. Images were acquired using a Zeiss Axiocam 305 color (Carl Zeiss Microscopy GmbH, Germany).

qRT-PCR

Tissue was homogenized using a mortar and pestle and RNA extracted by placing into QIAzol solution (Qiagen, UK). Tissue was lysed using a Qiagen Tissue lyser and RNA was extracted with miRNeasy® mini kit following the manufacturers protocol (Qiagen, UK). hPASMCs were scraped and also lysed using QIAzol solution. RNA was quantified using a nanodrop (ND-1000 spectrophotometer (Thermo-Fisher, UK). RNA was reverse transcribed to cDNA using the TaqMan™ reverse transcription kit (Applied Biosystems). Semi-quantitative real-time PCR was performed using an Applied Biosystems Viia 7 real-time PCR system. Specific dual labelled TaqMan™ primer-probe sets were purchased from Thermo-Fisher, UK and are shown in Table S1. Results are expressed as a ratio to a reference gene using the $2^{-\Delta Ct}$ comparison method.

Western blotting

Whole lung rat samples were homogenized and lysed in RIPA buffer (Sigma, UK) containing HALT protease and phosphatase inhibitors (Thermo-Fisher, UK). hPASMCs were lysed in ice-cold 1% (v/v) lauryl maltoside/PBS (Abcam, UK) containing HALT protease and phosphatase inhibitors. Protein concentrations were determined using BCA assay (Thermo-Fisher, UK). 20µg of protein was loaded for whole lung lysates, for protein identification by SDS-PAGE and immunoblotting. Protein expression was quantitated in immunoblots probed the relevant antibody by overnight incubation at 4°C. Antibodies used are shown in Table S2. Membranes were then incubated with anti-rabbit or anti-mouse secondary antibodies. Immunoblots were developed using Pierce™ ECL Western Blotting Substrate (Thermo-Fisher, UK) or EMD Millipore Immobilon™ Western Chemiluminescent HRP Substrate (ECL) (Fisher Scientific) and normalized to beta actin (Sigma, A5441) or alpha tubulin (Abcam, ab4074). In some instances, blots were cut and probed for two separate antibodies. This was only carried out where proteins of interest exhibited a large difference in molecular weight and where antibodies have been validated and shown to be specific for their target. Blots, where appropriate, may be stripped and re-probed using loading controls.

Body weights

End of study body weights were determined and shown in Figure S9.

Supplemental Tables

Table S1. Probe sets used within this study and Taqman™ assay ID details.

**Gene**	**Target**	**Assay ID**
NPPA (ANP)	Rat	Rn00664637
NPPB (BNP)	Rat	Rn00580641
CTGF	Rat	Rn01537279
FN1	Rat	Rn00569575
COL1A1	Rat	Rn01463848
COL3A1	Rat	Rn01437681
TGFβ	Rat	Rn00572010
GATA4	Rat	Rn01530459
IL6	Rat	Rn01410330
TNFα	Rat	Rn99999017
CCL5	Rat	Rn00579590
B2M	Rat	Rn00560865
GAPDH	Rat	Rn01775763
miR-96	Rat/Human	RT000186
U87	Rat/Human	RT001712
miR-16	Rat/Human	RT000391

Table S2. Antibodies and dilutions used for Western blotting

Antibody	Source	Supplier (catalogue #)	Dilution
5HT1BR	Rabbit	Abcam (13896)	WB 1:500
BMPR2	Mouse	BD Bioscience (612292)	WB 1:500
β-actin	Mouse	Sigma (A5441)	WB 1:5000
α-tubulin	Rabbit	Abcam (ab4074)	WB 1:12000

Supplemental figures and legends

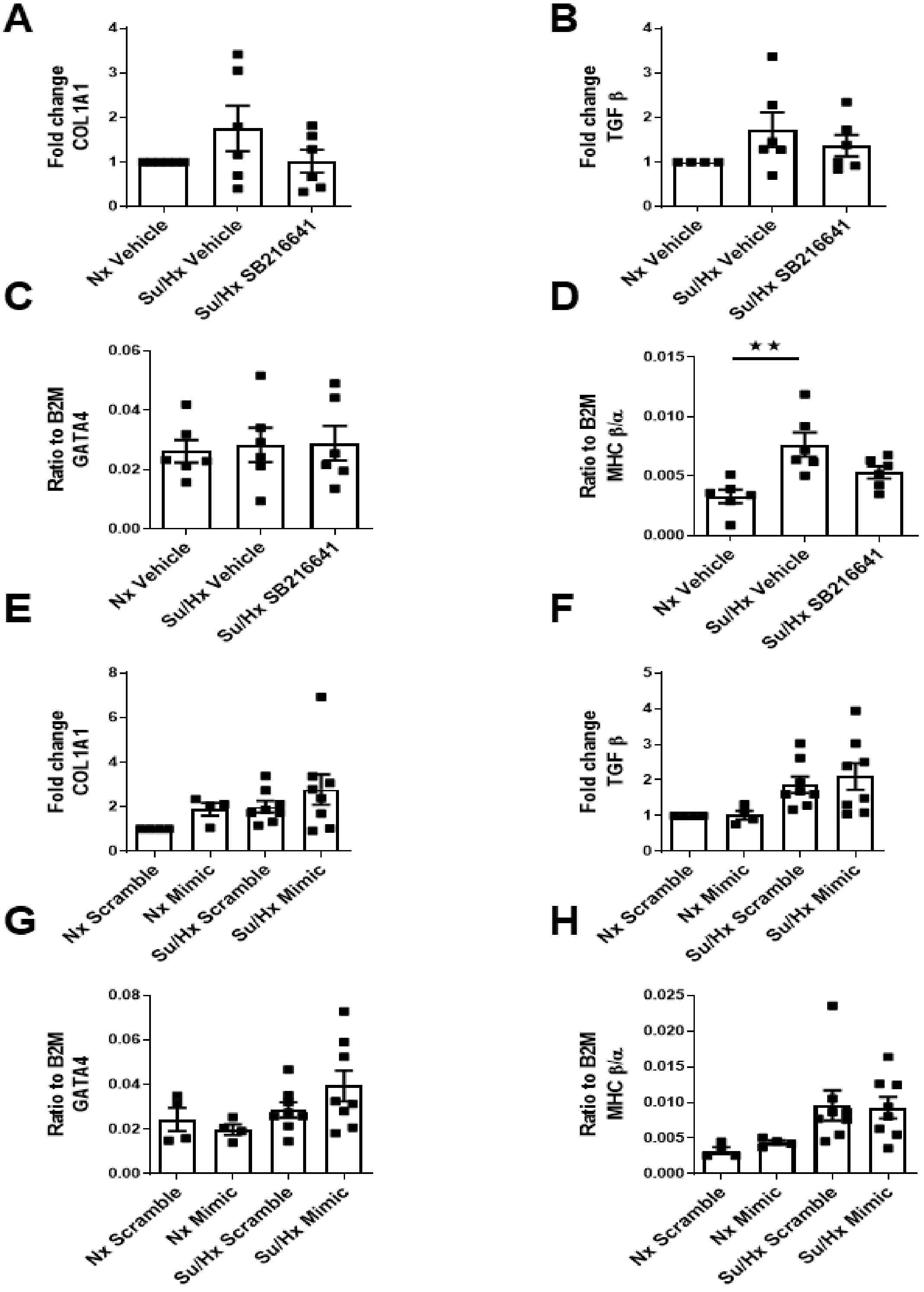

Figure S1. mRNA transcript expression of hypertrophic and fibrotic markers in the right ventricle. Collagen 1A1 (COL1A1) (A), Transforming growth factor beta

(TGFβ) (B), GATA binding protein 4 (GATA4) (C) and myosin heavy chain-β/α (β/α-MHC) (D) expression in RV tissue from normoxic (nx) vehicle and sugen/hypoxic (Su/Hx) ± SB216641 treated rats. COL1A1 (E), TGFβ (F), GATA 4 (G) and β/α-MHC (H) in normoxic miR96 mimic-treated vs sugen/hypoxic scramble sequence-treated and sugen/hypoxic miR96 mimic-treated rats. Error bars indicate mean ± SEM. One-way ANOVA with post-hoc Tukey's was used to assess statistical significance.

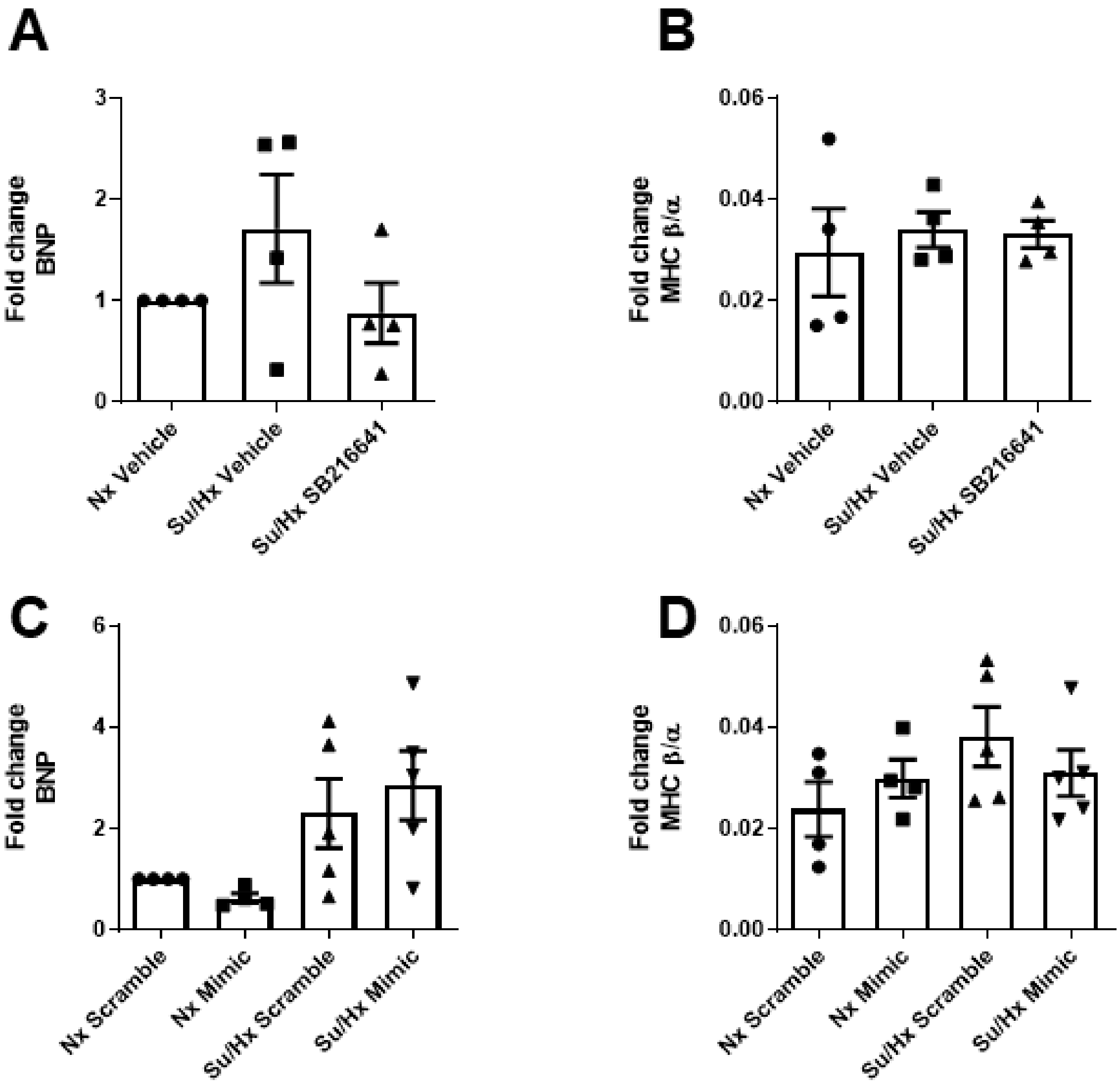

Figure S2. mRNA transcript expression of hypertrophic markers in the left ventricle. BNP (A) and β/α-MHC (B), in LV tissue from normoxic (Nx) vehicle (veh) and sugen/hypoxic (Su/Hx) and SB216641/vehicle treated rats. BNP (C) and β/α-MHC (D) expression in LV tissue from normoxic scramble sequence and normoxic miR96 mimic-treated vs sugen/hypoxic scramble sequence and sugen/hypoxic miR96-mimic treated rats. Error bars indicate mean ± SEM. One-way ANOVA with post-hoc Tukey's was used to assess statistical significance ★ = $p < 0.05$ (n=4-5).

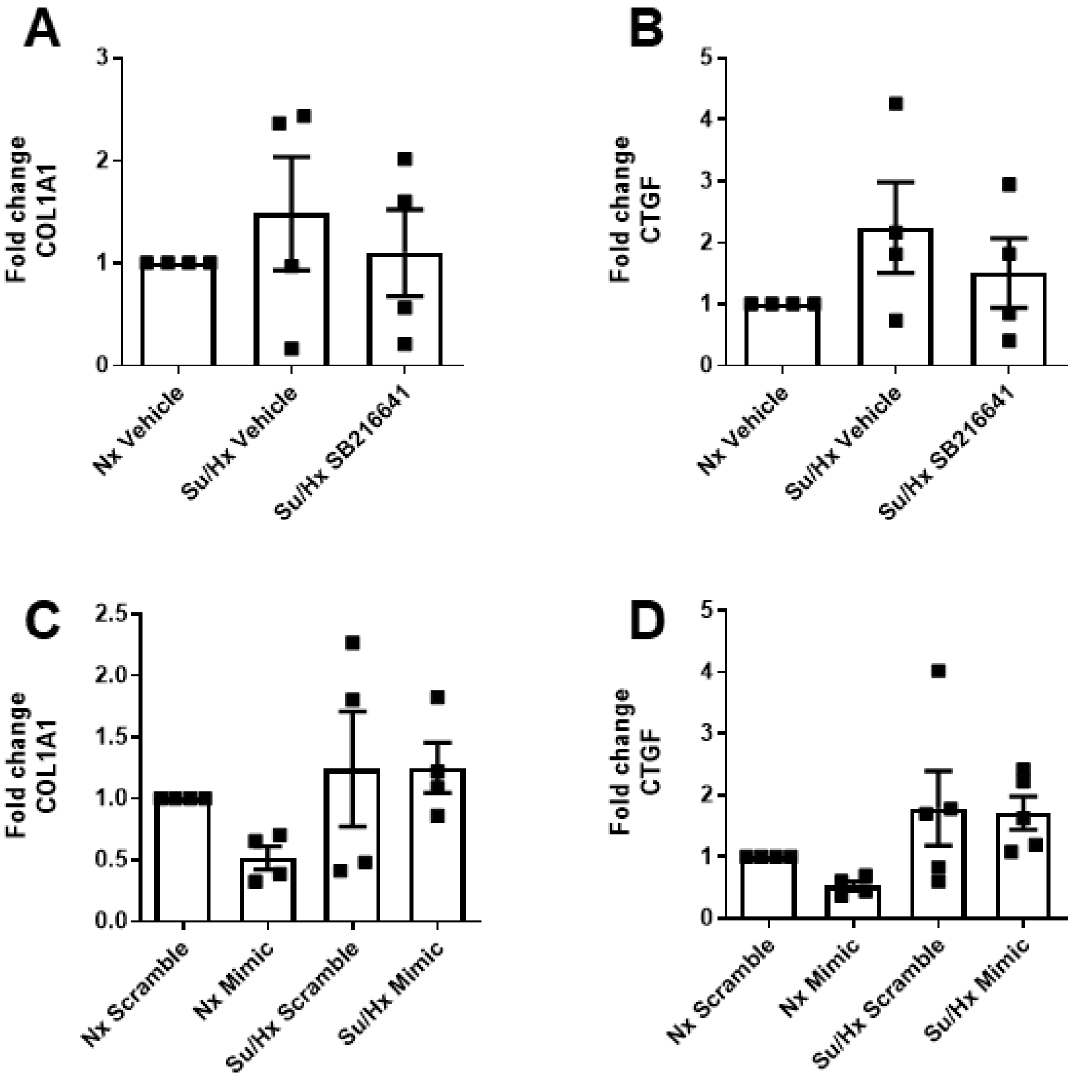

Figure S3. mRNA transcript expression of fibrotic markers in the left ventricle. COL1A1 (A) and CTGF (B) in LV tissue from normoxic (Nx) vehicle and sugen/hypoxic (Su/Hx) and SB216641/vehicle-treated rats. COL1A1 (C) and CTGF (D) in LV tissue from normoxic scramble sequence-treated and normoxic miR96 mimic –treated vs sugen/hypoxic scramble sequence-treated and sugen/hypoxic miR96 mimic-treated rats. Error bars indicate mean ± SEM. One-way ANOVA with post-hoc Tukey's was used to assess statistical significance (n=4-5).

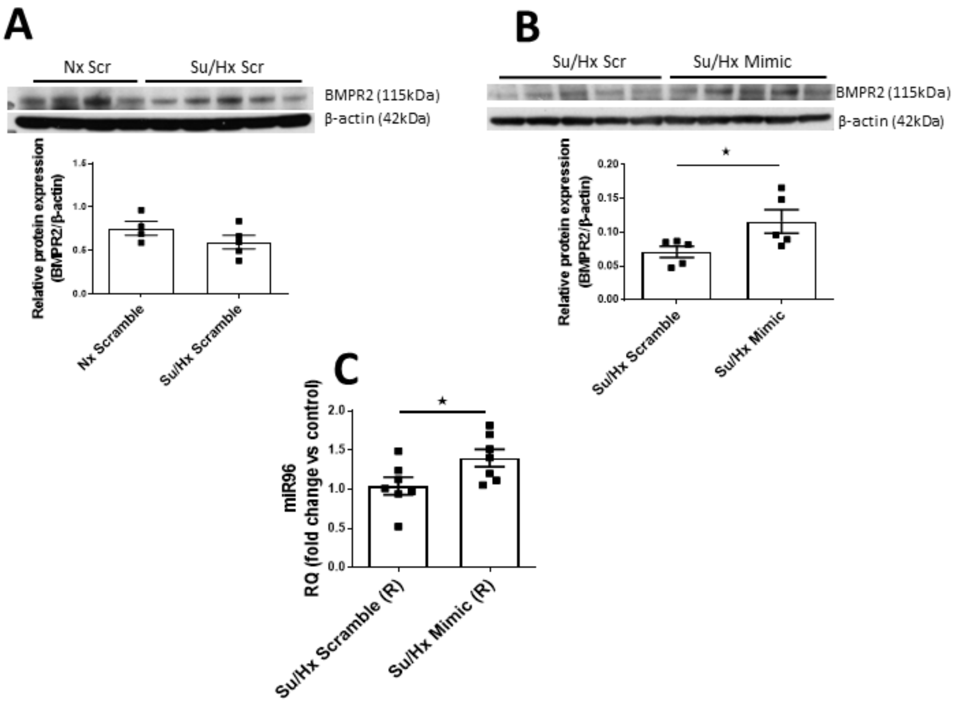

Figure S4. BMPR2 expression in Sugen-Hypoxic rat lung in presence of scramble sequence control or miR96 mimic. Representative Western Blot of BMPR2 protein expression in normoxic (Nx) scramble sequence control vs sugen/hypoxic (Su/Hx) control with densitometry analysis (A). Representative Western Blot of BMPR2 protein expression in Su/Hx scramble sequence control vs Su/Hx miR96 mimic with densitometry analysis (B). miR96 mRNA expression in the lungs of sugen/hypoxic (Su/Hx) scramble sequence and sugen/hypoxic miR96-dosed rats three weeks after last dose of miR96 (R) (C). Error bars indicate mean ± SEM. n=4-5 for each group in A, B. n=6-7 for C. Statistical significance was determined by students unpaired t-test (two-tailed). ★ $p<0.05$.

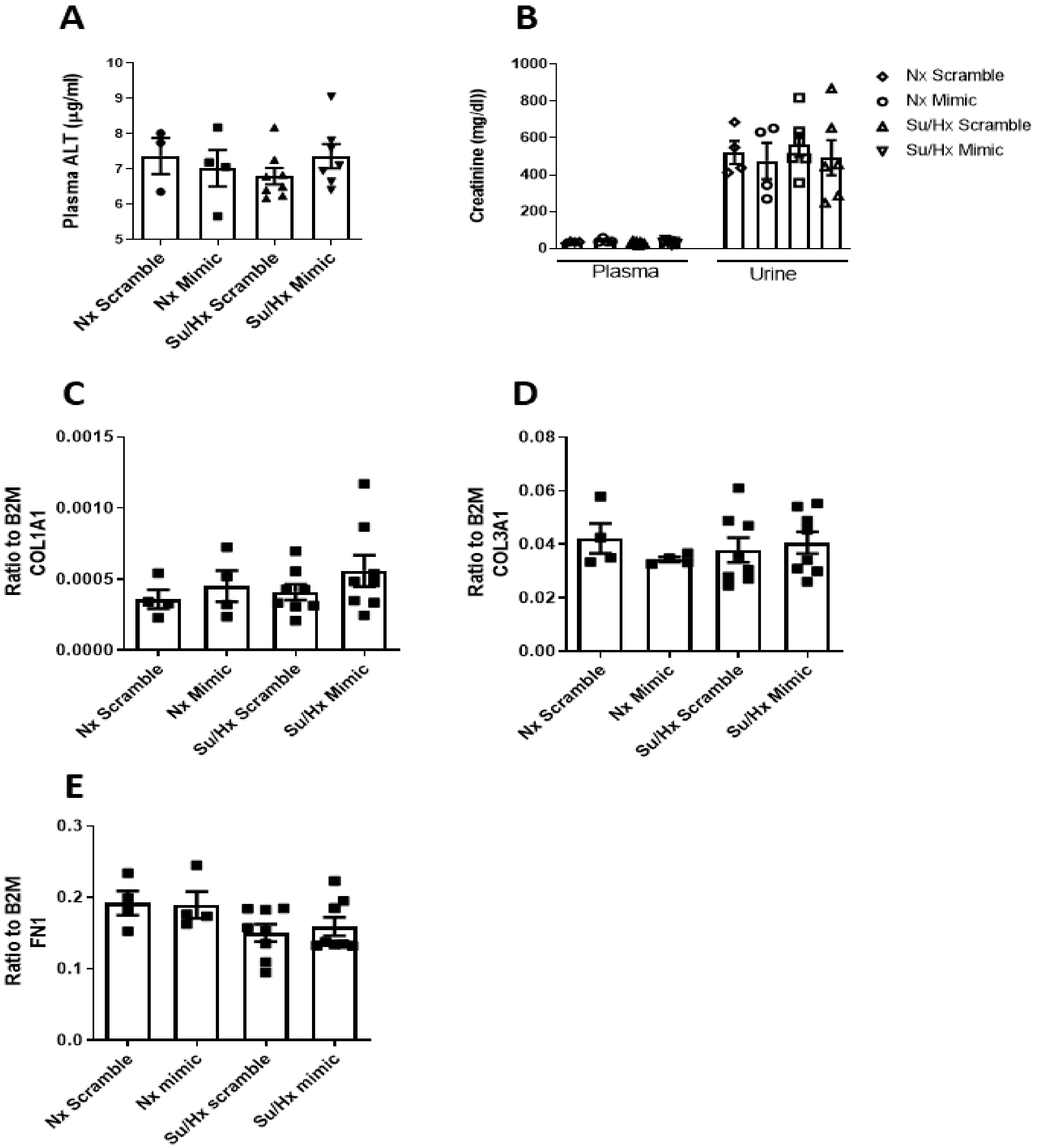

Figure S5. Effects of the miR-96 mimic on the liver and kidney. Plasma ALT levels (A) and plasma and urine creatinine levels (B) in normoxic (Nx) scramble sequence and normoxic miR96 mimic –treated vs sugen/hypoxic (Su/Hx) scramble sequence and Su/Hx miR96 mimic-treated rats. Col1a1 (C), Col3a1 (D) and FN1 (E) mRNA expression in the liver. Error bars indicate mean ± SEM. One-way ANOVA with post-hoc Tukey's was used to assess statistical significance (n=4-8).

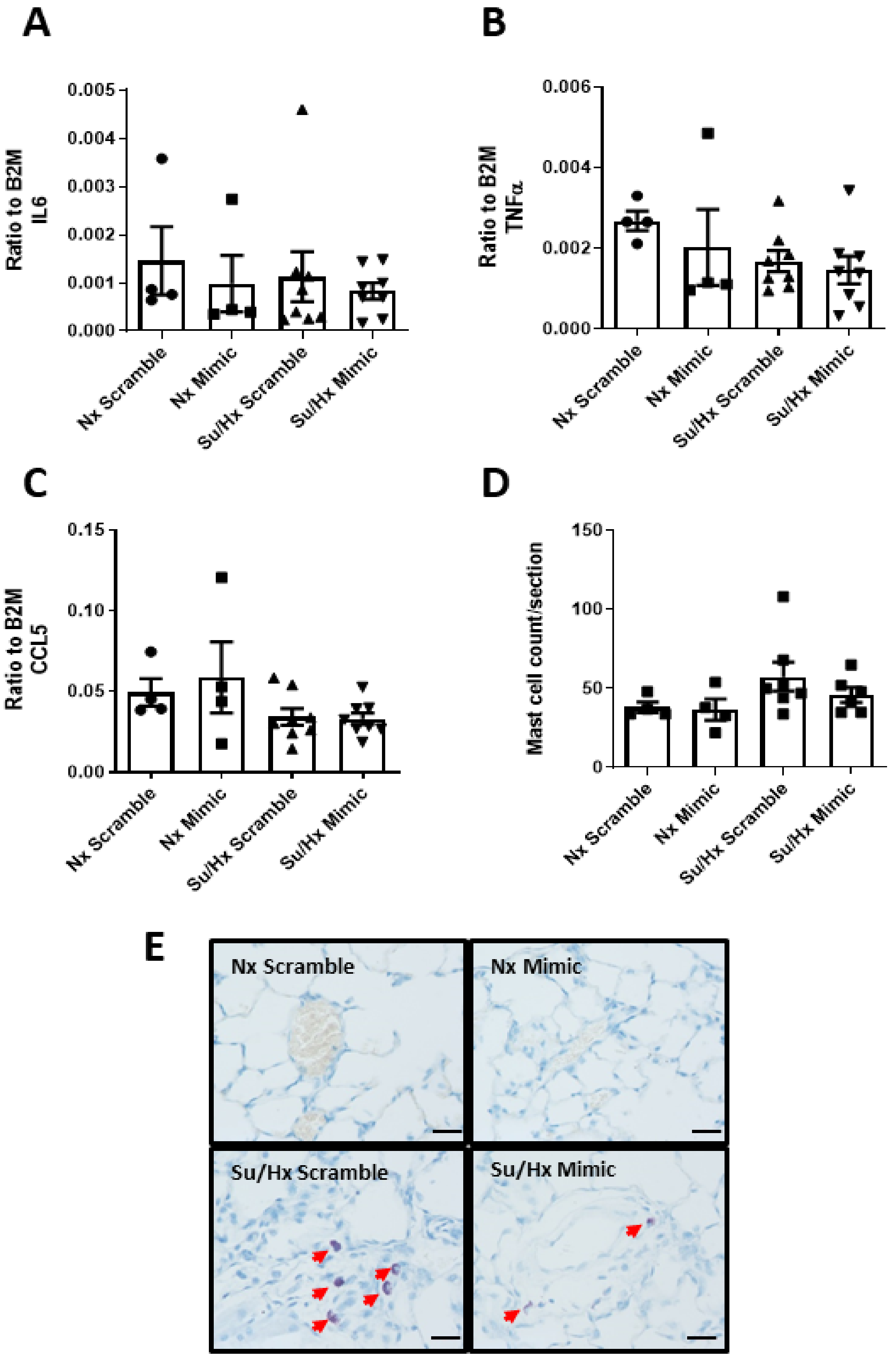

Figure S6. Effects of the miR-96 mimic on inflammation in the lung. IL6 (A), TNFα (B) and CCL5 (C) mRNA levels in lung tissue from normoxic (Nx) scramble sequence-

treated and normoxic miR96 mimic-treated vs sugen-hypoxic (Su/Hx) scramble sequence-treated and sugen-hypoxic miR96 mimic-treated rats. Mast cell number per lung section from normoxic scramble sequence-treated and normoxic miR96 mimic-treated vs sugen-hypoxic scramble sequence-treated and sugen-hypoxic miR96 mimic-treated rats (D) and representative images from each group (E). Arrows indicate mast cells. Error bars indicate mean ± SEM. One-way ANOVA with post-hoc Tukey's was used to assess statistical significance (n=4-8). Scale bars indicate 20μm.

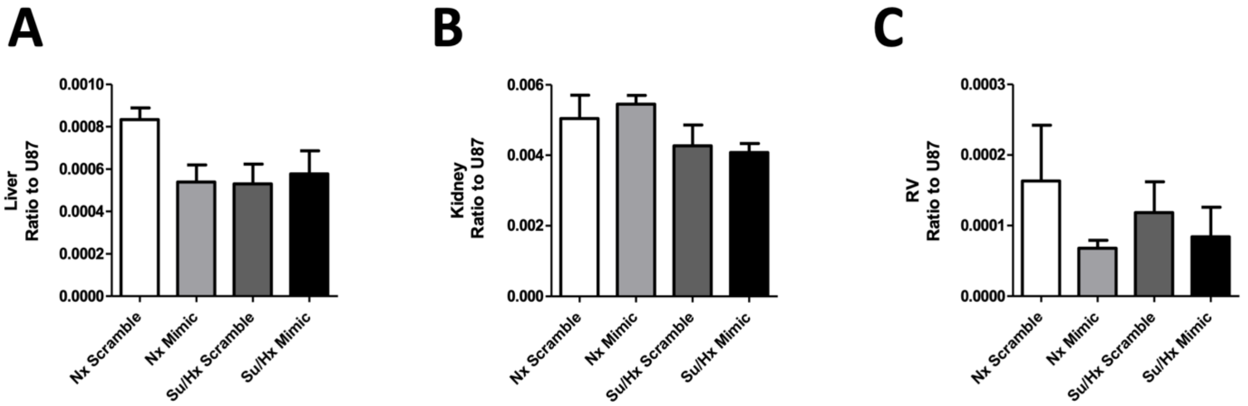

Figure S7. Expression of miR96 in liver, kidney and right ventricle (RV).

Expression of miR96 in liver (A), kidney (B), RV tissue (C) from normoxic (Nx) scramble sequence and normoxic miR96 mimic-treated vs sugen-hypoxic (Su/Hx) scramble sequence-treated and sugen-hypoxic miR96 mimic treated rats. Error bars indicate mean ± SEM. One-way ANOVA with post-hoc Tukey's was used to assess statistical significance (n=4-8).

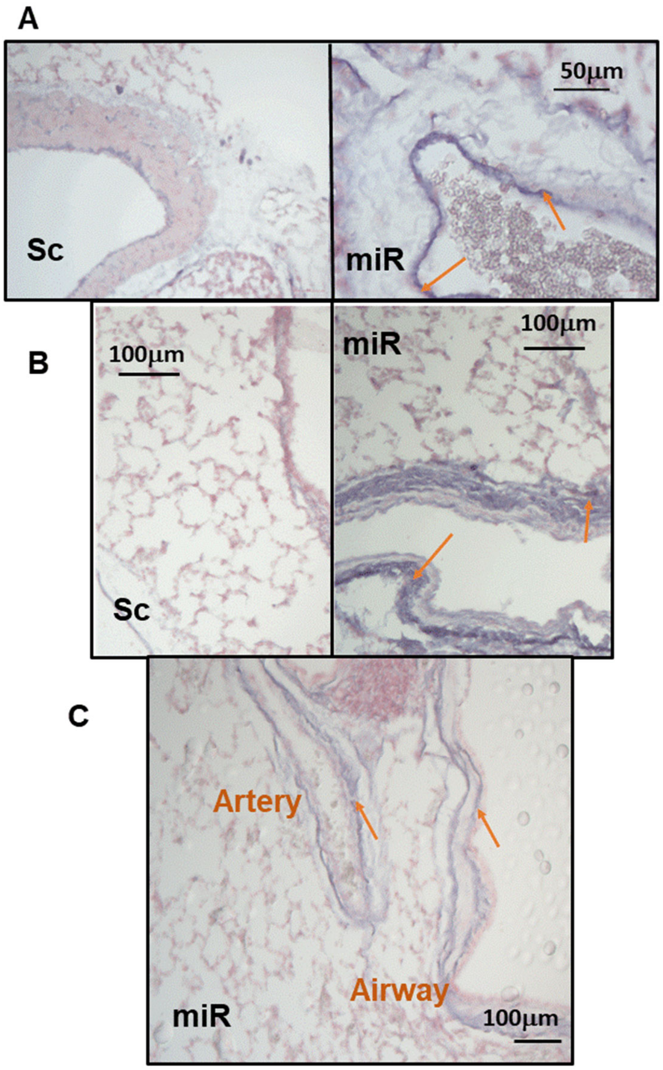

Figure S8. Expression of miR96 in large proximal pulmonary arteries and airway. MiR96 expression in large proximal pulmonary arteries (A,B) of sugen-hypoxic scramble sequence (Sc) and sugen-hypoxic miR96 mimic treated rats (miR). miR96 expression in a large proximal pulmonary artery (Artery) and adjacent airway (Airway) of sugen-hypoxic miR96 mimic treated rats (C). Positive miR96 is indicated by purple staining by arrows.

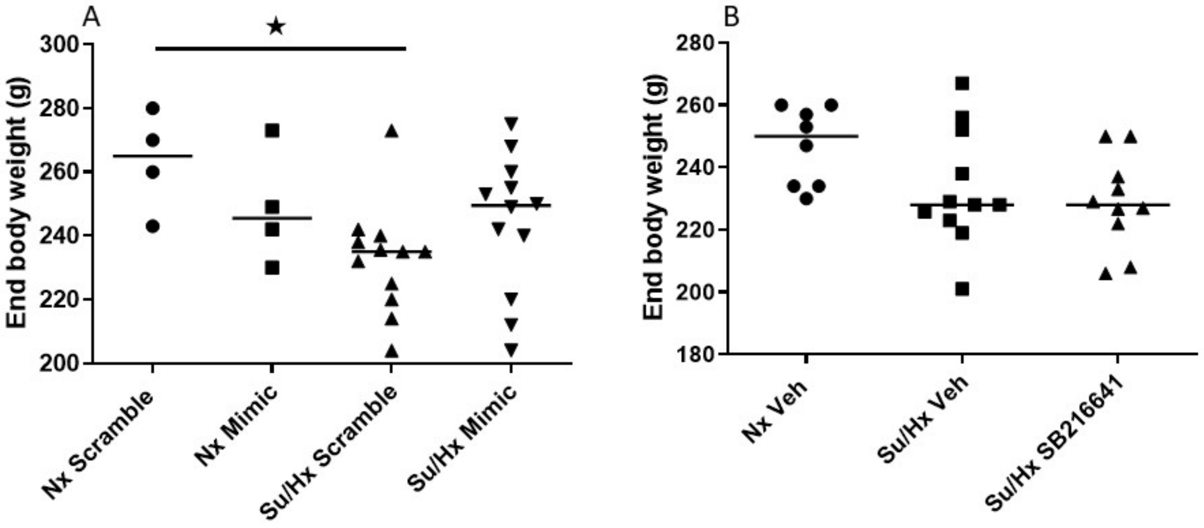

Figure S9. End of study body weights

(A) Normoxic (Nx) scramble sequence and normoxic miR96 mimic-treated vs sugen-hypoxic (Su/Hx) scramble sequence-treated and sugen-hypoxic miR96 mimic treated rats. (B) normoxic (Nx) vehicle and sugen/hypoxic (Su/Hx) and SB216641/vehicle-treated rats.
