# Supplementary material for: Direct Delivery of MicroRNA96 to the Lungs Reduces Progression of Sugen/Hypoxia-Induced Pulmonary Hypertension in the Rat
Source: Mol Ther Nucleic Acids. 2020 Sep 6;22:396–405. doi: 10.1016/j.omtn.2020.09.002 (PMC7533346; doi:10.1016/j.omtn.2020.09.002)
Supplement: Document S2. Article plus Supplemental Information [file mmc2.pdf]

Molecular Therapy
Nucleic Acids
Original Article

Direct Delivery of MicroRNA96 to the Lungs Reduces Progression of Sugen/Hypoxia-Induced Pulmonary Hypertension in the Rat

Craig K. Docherty,[1,2] Nina Denver,[1] Simon Fisher,[2] Margaret Nilsen,[1,2] Dianne Hillyard,[2] Rebecca L. Openshaw,[1] Hicham Labazi,[1] and Margaret R. MacLean[1,2]

[1]Strathclyde Institute of Pharmacy and Biological Sciences, University of Strathclyde, Glasgow G4 0RE, Scotland; [2]Institute of Cardiovascular and Medical Sciences, College of Medical Veterinary and Life Sciences, University of Glasgow, Scotland

The 5HT1B receptor (5HT1BR) contributes to the pathogenic effects of serotonin in pulmonary arterial hypertension. Here, we determine the effect of a microRNA96 (miR96) mimic delivered directly to the lungs on development of severe pulmonary hypertension in rats. Female rats were dosed with sugen (30 mg/kg) and subjected to 3 weeks of hypobaric hypoxia. In normoxia, rats were dosed with either a 5HT1BR antagonist SB216641 (7.5 mg/kg/day for 3 weeks), miR96, or scramble sequence (50 μg per rat), delivered by intratracheal (i.t) administration, once a week for 3 weeks. Cardiac hemodynamics were determined, pulmonary vascular remodeling was assessed, and gene expression was assessed by qRT-PCR, and *in situ* hybridization and protein expression were assessed by western blot and ELISA. miR96 expression was increased in pulmonary arteries and associated with a downregulation of the 5HT1BR protein in the lung. miR96 reduced progression of right ventricular systolic pressure, pulmonary arterial remodeling, right ventricular hypertrophy, and the occurrence of occlusive pulmonary lesions. Importantly, miR96 had no off-target effects and did not affect fibrotic markers of liver and kidney function. In conclusion, direct delivery of miR96 to the lungs was effective, reducing progression of sugen/hypoxia-induced pulmonary hypertension with no measured off-target effects. miR96 may be a novel therapy for pulmonary arterial hypertension, acting through downregulation of 5HT1BR.

INTRODUCTION

Pulmonary arterial hypertension (PAH) is a chronic condition defined by vascular remodeling and complex vascular lesion formation arising from accelerated proliferation of pulmonary endothelial, smooth muscle, and fibroblast cells.[1] Increased activation of the serotonergic system has been implicated in the development and progression of pulmonary hypertension (PH).[2,3] This includes increased activation of the serotonin transporter (SERT) and the serotonin 1B receptor (5HT1BR), as well as the synthesis of serotonin via tryptophan hydroxylase 1 (TPH1).[2,4] Synthesis of serotonin via TPH1 has been associated with both experimental and clinical PAH,[5–7] and TPH1 inhibitors can reverse experimental PH.[8] Overexpression of the SERT in female mice leads to increased basal right ventricular (RV) systolic pressure (RVSP) and renders the mice more susceptible to hypoxia-induced PH.[9] Therefore, inhibition of the serotonin pathway may have beneficial effects in the treatment of PAH.

It is the 5HT1BR that mediates serotonin-induced contraction of conduit and resistance human pulmonary arteries[10,11] and serotonin-induced proliferation of female human pulmonary arterial smooth muscle cells (hPASMCs).[12] 5HT1BR antagonism or knockout can prevent hypoxia-induced PH in rodents.[13] MicroRNAs (miRNAs) are short, non-coding nucleic acid sequences that can affect downstream systems by directing degradation of mRNA transcripts or silencing mRNA translation. Several miRNAs have subsequently been implicated in the development of PAH,[14] and many are involved in serotonergic signaling in other systems of the body.[15,16] miRNA96 (miR96) is the only known miRNA that targets the 5HT1BR,[12] and miR96 levels are decreased in female PAH patient hPASMCs when compared to non-PAH controls.[12] This is accompanied by increased expression of the 5HT1BR. Both increased 5HT1BR expression and proliferation can be inhibited by a miR96 mimic. Hence, decreased miR96 is likely to play a role in the increased serotonin/5HT1BR-induced hyper-proliferation observed in female PAH patient hPASMCs. This suggests that miR96 may offer a novel therapeutic approach to PAH, selectively targeting the serotonin receptor responsible for pathogenic effects. The mechanisms of serotonin/5HT1BR-induced proliferation of hPASMCs are well documented now by ourselves and others,[12,17–19] so there was no requirement to investigate this further in this study. The novelty of this study is that we deliver the miR96 directly to the lung and evaluate the ability of lung-delivered miR96 to reverse progression of PH in the robust sugen/hypoxia rat model. We also examine the effects of miR96 on liver, kidney, and cardiac function and fibrosis. To compare

Received 3 June 2020; accepted 3 September 2020;
https://doi.org/10.1016/j.omtn.2020.09.002.

Correspondence: Margaret R. MacLean, Strathclyde Institute of Pharmacy and Biomedical Sciences, University of Strathclyde, HW406, The Hamnett Wing, 161 Cathedral Street, Glasgow G4 0RE, Scotland.
E-mail: mandy.maclean@strath.ac.uk

 © 2020 The Authors.
This is an open access article under the CC BY license (http://creativecommons.org/licenses/by/4.0/).

the effects of the miR96 mimic with those of a known 5HT1BR antagonist, we compared the hemodynamic and off-target effects with those of the 5HT1BR antagonist SB216641. In addition, we determined longer term effects of lung-delivered miR96 and the effects of miR96 on liver and kidney function. This provides novel and unique pre-clinical data to inform potential translational studies.

RESULTS

Effects of miR96 on Pulmonary Hemodynamics in the Sugen/Hypoxic Rat

RVSP was increased in the sugen/hypoxic rats, and miR96 reduced RVSP by ∼30% (Figure 1A). RV hypertrophy (RVH) was doubled in the sugen/hypoxic rats, and miR96 delivery reduced RVH by ∼22% (Figure 1B). The percentage of remodeled pulmonary arteries was markedly increased in the sugen/hypoxic scramble sequence control group versus the normoxic control group, and miR96 reduced this by ∼40% (Figure 1C). The number of occluded vessels was not significantly increased in the miR96 treated animals (Figure 1D). Representative images of remodeled arteries are shown in Figure 1E, and images of occluded arteries stained for endothelial cells are shown in Figure 1F. Three weeks after the final dose of miR96, the hemodynamic and histological analyses were repeated in a further group of rats to investigate whether there was any recovery or longer term effects of miR96. RVSP had recovered to pre-miR96 levels, as had RVH, and there was no further effect of miR96 (Figures 1A and 1B). The percentage of remodeled vessels had recovered slightly, but the inhibitory effect of miR96 remained after 3 weeks (Figure 1C).

Effects of a 5HT1BR Antagonist on Pulmonary Hemodynamics in the Sugen-Hypoxic Rat

SB216641 reduced RVSP and RVH by ∼38% and ∼20%, respectively, in the sugen/hypoxic rats (Figures 2A and 2B). SB216641 had no significant effect on the total percentage of remodeled vessels (Figure 2C) but reduced the percentage of occluded lesions in the sugen/hypoxic rats (Figure 2D). Representative images of remodeled arteries are shown in Figure 2E, and images of occluded arteries stained for endothelial cells are shown in Figure 2F.

Effects of miR96 and 5HT1BR Antagonism on Markers of Cardiac Hypertrophy and Fibrosis

B-type natriuretic peptide (*BNP*; *NPPB*) gene expression was increased in the sugen/hypoxic right ventricle (Figures 3A and 3B). After SB216641 administration, *BNP* mRNA levels were no longer significantly higher than in the vehicle controls (Figure 3A). BNP levels were not affected by miR96 (Figure 3B). RV mRNA levels of connective tissue growth factor (*CTGF*) were significantly increased in the sugen/hypoxic rats (Figures 3C and 3D), and mRNA levels were normalized by the 5HT1BR antagonist (Figure 3C) but were unaffected by miR96 (Figure 3D).

Neither miR96 nor SB216641 affected RV expression of collagen 1A1 (COL1A1), transforming growth factor-β (*TGFβ*), GATA binding protein 4 (*GATA4*), or myosin heavy chain-β/α (*MHCβ/MHCα*) (Figures S1A–S1H). *BNP* or *MHCβ/MHCα* mRNA ratios (Figures S2A–S2D) and *COL1A1* and *CTGF* expression (Figures S3A–S3D) were unaffected in the sugen/hypoxic rat in the left ventricle.

Effects of miR96 and a 5HT1BR Antagonist on RV Function

RV contractility was measured by the maximum derivative of pressure/derivative of time (dP/dt max), which was increased in the sugen/hypoxic rats; however, this was not affected by SB216641 (Figure 4A) but was reduced by miR96 (Figure 4B). Ventricular lusitropy (minimum derivative of pressure/derivative of time; dP/dt min) was reduced by SB216641 (Figure 4C) and by miR96 (Figure 4D). RV load was increased in the sugen/hypoxic rats (Figure 4E). This was not affected by SB216641 (Figure 4E), but progression was reduced by miR96 (Figure 4F).

Ejection fraction was unaffected by miR96 or SB216641 (Figures 5A and 5B). RV loop power was increased in the sugen/hypoxic rat (Figures 5C and 5D), and this progression was prevented by miR96 (Figure 5D). Heart rate was unaffected by miR96 or SB216641 (Figures 5E and 5F).

Effects of miR96 on 5HT1BR Expression in the Lung

miR96 mRNA expression was increased by ∼33% in the lungs after administration of miR96 (Figure 6A). *In situ* analysis of miR96 expression demonstrated that miR96 uptake was not uniform across the lung but appeared in discrete regions. Figure 6B demonstrates that miR96 expression was localized to the vascular wall of small pulmonary arteries, and this was not observed after administration of the scramble sequence. There was an increase in 5HT1BR expression within the sugen/hypoxic rat lungs (Figure 6C), and miR96 decreased 5HT1BR expression in the sugen/hypoxic rat lung (Figure 6D).

BMPR2 protein expression was not altered in the sugen/hypoxic scramble sequence group, compared to the normoxic controls (Figure S4A), but was increased by miR96 in the sugen/hypoxic rat lung (Figure S4B). Three weeks after the last dose of miR96, increased miR96 expression was still measurable in the lungs of the sugen/hypoxic rats (Figure S4C), although the increase was only ∼21% compared to ∼33% 3 weeks before (Figure 6A).

Effects of miR96 on Liver and Kidney Function and Inflammatory Responses in the Lung

We investigated possible renal and hepatic toxicity in the miR96-treated animals. An increase in plasma alanine aminotransferase (ALT) would indicate liver damage or inflammation and elevated creatinine levels signifiy impaired kidney function. miR96 did not have any adverse effects on rat plasma ALT levels (Figure S5A) or on creatinine levels in rat plasma and rat urine (Figure S5B). miR96 also had no effects on fibrotic mRNA markers *COL1A1*, *COL3A1*, and fibronectin (*FN1*) in the liver (Figures S5C–S5E). Similarly, miR96 had no effect on the gene expression of the inflammatory markers interleukin-6 (*IL6*) and tumor necrosis factor α (*TNFα*) or the chemokine ligand 5 (*CCL5*) in the lung (Figures S6A–S6C). There was also no effect of miR96 on mast cell accumulation in the lung (Figures S6D and S6E).

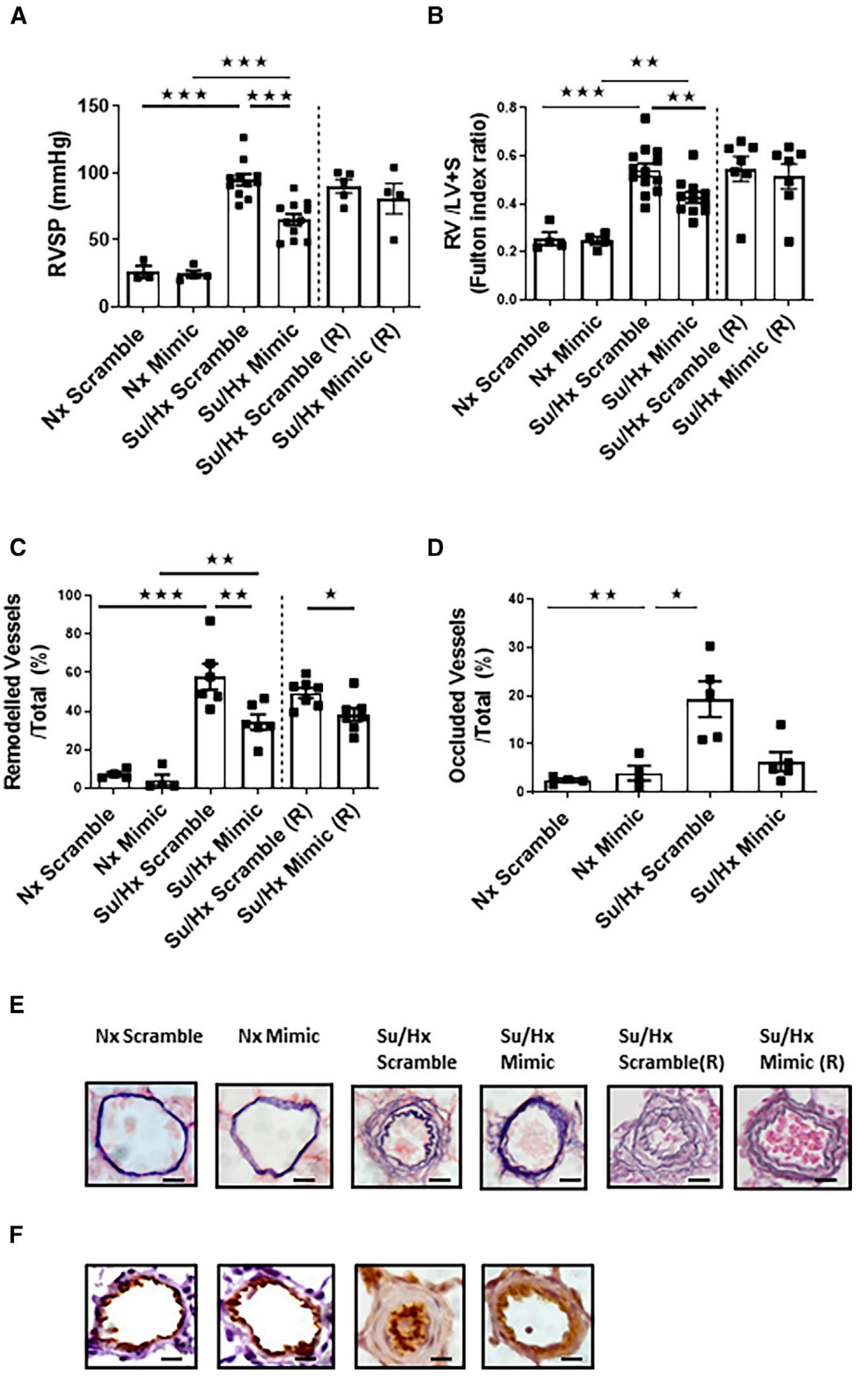

Figure 1. Hemodynamic and Hypertrophic Indicators in the Sugen-Hypoxic Rat Model in the Presence of miR96

(A and B) Right ventricular (RV) systolic pressure (RVSP) (A) and RV hypertrophy, as measured by RV weight/left ventricular (LV) weight + septum weight (B) after normoxia (Nx) or sugen/hypoxia (Su/Hx) with administration of scramble sequence (Scramble) or miR96-mimic (i.t.) (Mimic). n = 4. (C and D) Number of remodeled vessels (C) and occluded vessels (D) after 3 weeks of hypoxia and 3 weeks of Sugen (30 mg/kg) + scramble sequence or miR96-mimic (i.t.) or in Nx conditions. Su/Hx scramble sequence (R) and Su/Hx mimic (R) indicate data (n = 4) taken 3 weeks after the last dose of scramble sequence or mimic. (E and F) Representative images of elastin-picrosirius-red-stained (E) and Von-Willebrand-stained (F) distal pulmonary artery endothelial cells. Error bars indicate mean ± SEM. n = 3–4 for Nx, and n = 6–13 for Hx. Statistical significance was determined by one-way ANOVA with Tukey's post hoc test. ★$p < 0.05$; ★★$p < 0.01$; ★★★$p < 0.001$. Scale bars, 20 μm.

miR96 Expression in Other Tissues, Large Pulmonary Arteries, and Airways

Lung delivery of miR96 did not affect miR96 expression in liver (Figure S7A), kidney (Figure S7B), or RV tissue (Figure S7C). *In situ* localization also shows that miR96 expression was present in the smooth muscle and endothelium of large proximal pulmonary arteries and the smooth muscle of airways of miR96-treated rats (Figures S8A–S8C).

DISCUSSION

This is the first study to demonstrate that miR96 can be delivered directly to the lungs in a model of occlusive PH with increased expression in pulmonary arteries inducing therapeutic effects. This is associated with miR96 targeting 5HT1BR and decreasing lung expression, as we demonstrated previously.[12] We have determined secondary therapeutic effects of lung-delivered miR96 on RV function and shown that transfection was not associated with increases in inflammatory markers or lung fibrosis and that liver and kidney function was not affected. In addition, we demonstrate therapeutic effects of a systemically delivered 5HT1BR antagonist in this model for the first time.

We assessed the effectiveness of direct delivery of miR96 to the lungs in the sugen/hypoxic rat model of PH. In this model, after initial hypoxic exposure, the PH increases with time once the animals are exposed to normoxic conditions.[20] The model demonstrated elevated RVSPs (over 90 mmHg), profound RV hypertrophy, and fibrosis, as well as pulmonary vascular remodeling and occlusive lesions in the distal pulmonary arteries. RV contractility was elevated in the rat model, suggesting that this is a model of early-stage PH where the right ventricle has adapted to afterload with increased contractility. Consistent with being a model of PH, the cardiac effects were only observed in the right ventricle, with no changes in the left ventricle. It should be noted, however, that one limitation of this study is that hemodynamic parameters, including heart rate, are affected by anesthesia.

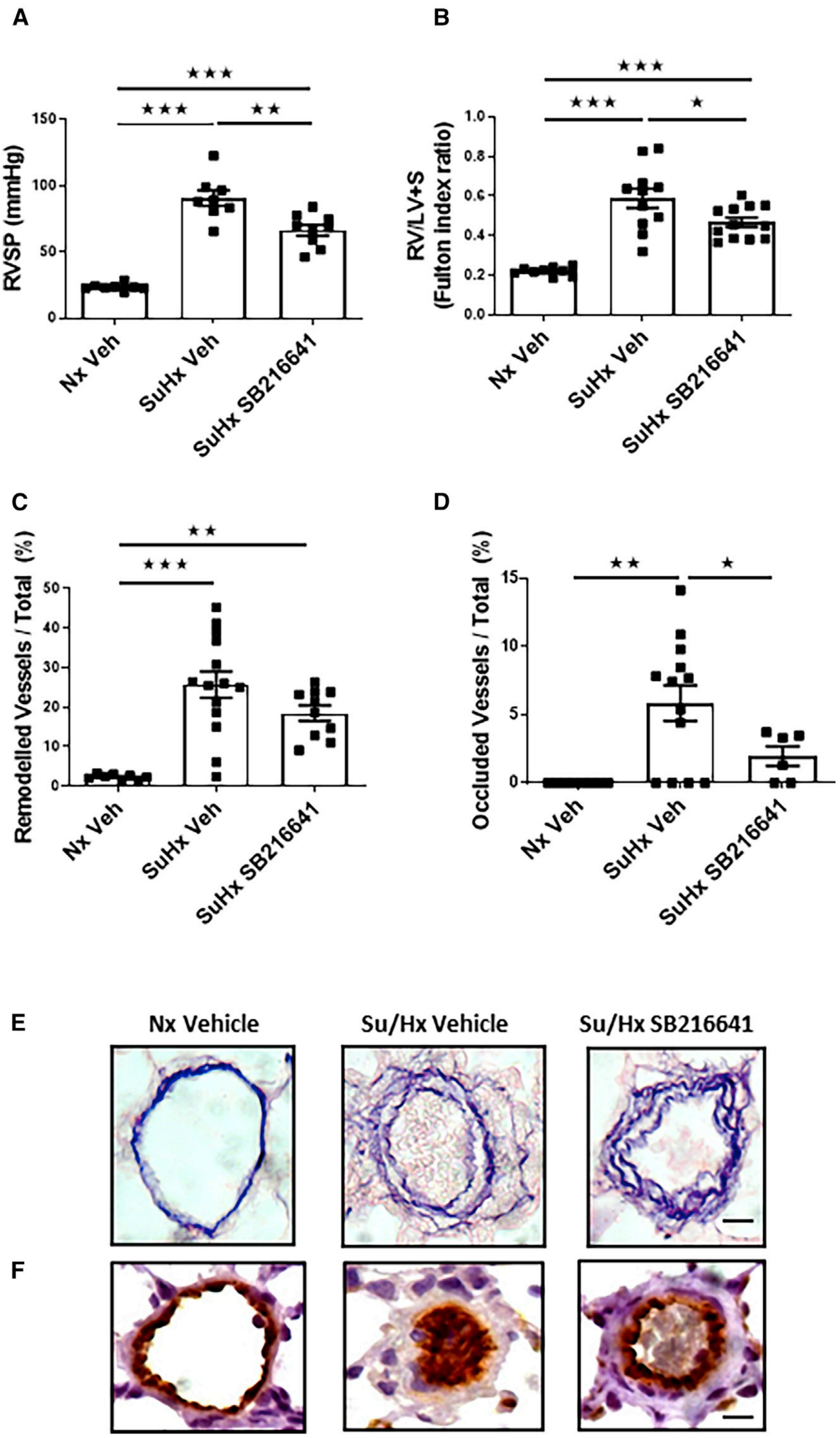

Figure 2. Hemodynamic and Hypertrophic Indicators in the Sugen-Hypoxic Rat Model in the Presence of the 5HT1BR Antagonist SB216641

(A and B) RVSP (A) and RV hypertrophy, as measured by RV weight/LV weight + septum weight (RV/LV+S) (B) in Nx vehicle (Veh)-treated group compared to Su/Hx Veh-treated and sugen-hypoxic antagonist-treated group. (C and D) Number of remodeled vessels (C) and occluded vessels (D) in Nx-Veh-treated group compared to Su/Hx Veh-treated and Su/Hx antagonist-treated group. (E and F) Representative images of elastin-picrosirius red-stained distal pulmonary arteries (E) and Von Willebrand-stained distal pulmonary arteries (F). Error bars indicate mean ± SEM; n = 4–11 for each group. Statistical significance was determined by one-way ANOVA with Tukey's post hoc test. *$p < 0.05$; **$p < 0.01$; ***$p < 0.001$. Scale bars, 20 μm.

After direct delivery of miR96 to the lung, as expected, we observed increased expression of miR96 in the large proximal pulmonary arteries and airways but also in the smooth muscle of small distal pulmonary arteries. The distribution of miR96 appeared in discrete areas of the lung, as it is distributed in one inhalation. This is similar to the situation for inhalation of bronchodilators and anti-inflammatory agents, where it has been shown that only 20% of drug is delivered, with the majority being swallowed.[21] The observation that, despite this, miR96 was effective suggests that this approach has much potential for development. Direct delivery of miR96 to the sugen/hypoxic rat lung also decreased 5HT1BR expression in small pulmonary arteries. This is consistent with our previous studies showing miR96 downregulates the 5HT1BR.[12] To date, miR96 is the only miRNA that has been identified as targeting *5HT1BR*. miR96 reduced the progression of pulmonary vascular remodeling and inhibited the development of occlusive pulmonary lesions. miR96 also reduced the progression of increases in RVSP and RVH. As we could not detect increased miR96 expression in RV tissue after i.t. administration, these therapeutic effects are secondary to improvements in the pulmonary circulation. BNP is released in response to changes in pressure inside the heart and is a marker of cardiac dysfunction.[22] While *BNP* gene expression was elevated in the right ventricle of the sugen/hypoxic rats, miR96 delivered to the lungs did not affect BNP gene expression in the right ventricle. Likewise, miR96 did not normalize elevated *CTGF* expression, a marker of cardiac fibrosis. This reflects the administration of miR96 directly to the lungs; however, it is possible that these adaptive changes might have resolved with longer term treatment with miR96, secondary to resolution of vascular changes in the pulmonary circulation.

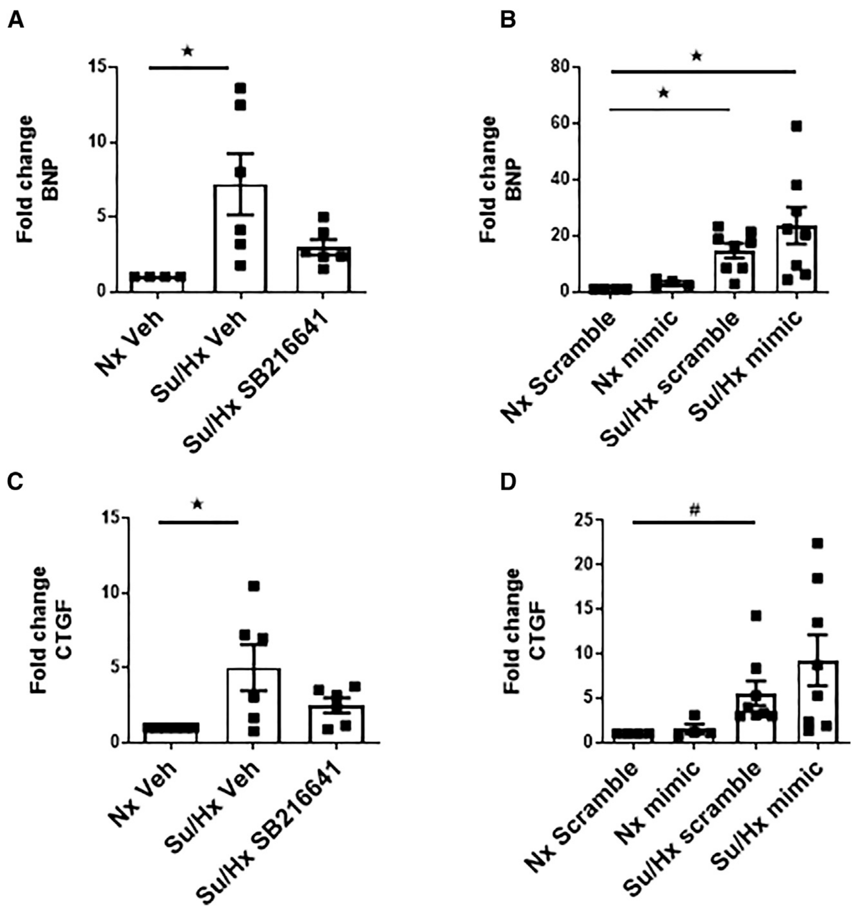

Figure 3. Effects of SB216641 and miR96 (Mimic) on mRNA Transcript Expression of Hypertrophic and Fibrotic Markers in the Right Ventricle: Expression in RV Tissue from Nx Veh and Su/Hx Rats

(A–D) BNP (*NPPB*): SB216641 (A); BNP: mimic (B); CTGF: SB216641 (C); and CTGF: mimic (D). Error bars indicate mean ± SEM. A one-way ANOVA with Tukey post hoc test was used. $^{\star}p < 0.05$; $^{\#}p < 0.05$, Student's unpaired t test. n = 4–8.

We wanted to determine the longer term effects of miR96 and so studied effects on the pulmonary circulation 3 weeks after miR96 administration. While there was still an increase in lung miR96 expression, this was reduced by 12%, compared with expression 3 weeks earlier. After 3 weeks, miR96 had no effects on RVSP, RVH, or pulmonary occlusions, but the inhibitory effects on pulmonary vascular remodeling were still evident. This is consistent with the vascular remodeling being the last index of PH to resolve in the hypoxic model of PH when rats are removed from hypoxia and placed in normoxia.[23] This would indicate that weekly maintenance doses of miR96 might be required therapeutically.

Right heart failure can arise from both contraction (inotropic) and relaxation (lusitropic) abnormalities. Previous studies have shown that adaptive responses in these indices are associated with PAH.[24] The therapeutic effects of miR96 on the pulmonary circulation caused a secondary reversal of RV contractility and RV load. The increased contractility is an adaptive response of the right ventricle, and reversal is consistent with a decrease in afterload. Consistent with this, RV load, or pulmonary effective arterial elastance (Ea) (measure of total RV afterload) was also reduced by miR96.

The orally administered 5HT1BR antagonist was also effective in reducing RVSP and RVH. Due to compound precipitation issues, this compound could not be delivered directly to the lungs. SB216641 reduced the pulmonary occlusive lesions in the rat model. This is consistent with previous studies where we demonstrated that a 5HT1BR antagonist and *HTR1B* knockdown protected mice from hypoxia-induced PH.[13] However, at this dose, SB216641 did not significantly reduce the percentage of remodeled pulmonary arteries in the sugen/hypoxic rat where ∼60% of vessels were remodeled. An alternative 5HT1BR antagonist did reverse remodeling in the hypoxic rat, where this was less severe (25%).[13] In this regard, therefore, miR96 was more effective than the 5HT1BR antagonist. This could have been due to increased reduction in receptor expression being more effective than competitive antagonism or because miR96 was having the additional therapeutic effects observed and discussed earlier. 5HT1BRs are Gi-coupled and are subject to significant pharmacological synergy,[25–28] which may overcome 5HT antagonism to a greater extent than a reduction in receptor number. The 5HT1BR antagonist also normalized *BNP* mRNA levels in the right ventricle, consistent with its having been administered orally. SB216641 reduced the adaptive increase in lusitropy (dP/dt min) but did not affect inotropy or RV load. This likely reflects its more moderate effects (versus miR96) in reversing RVSP and RV hypertrophy, as well as the lack of effects on the percentage of remodeled pulmonary arteries (hence, less reduction in afterload).

We previously demonstrated that female (not male) PAH patient hPASMCs overexpress the 5HT1BR and exhibit increased serotonin-induced proliferation, mediated by the 5HT1BR. Accordingly, transfection of these cells with miR96 both reduced the expression of the 5HT1BR and inhibited serotonin-induced proliferation.[12] The increased expression of the 5HT1BR in female PAH hPASMCs is likely to be due to the effects of estrogen, which can induce expression of the 5HT1BR in these cells.[29]

We and others have previously determined molecular mechanisms of 5HT1BR stimulation in the pulmonary circulation and in PASMCs,

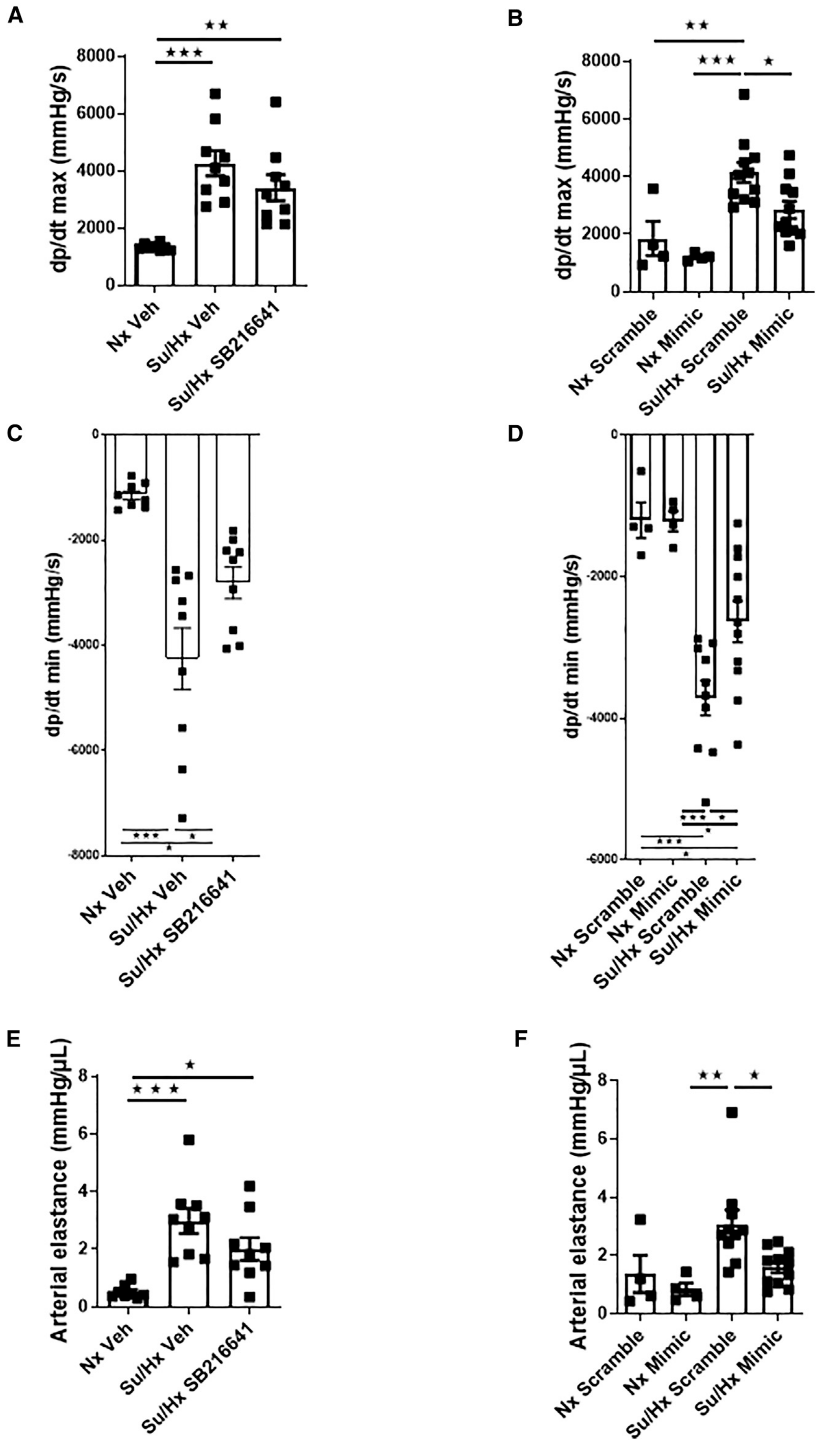

Figure 4. Hemodynamic Assessment of RV Function Su/Hx Rat Model in the Presence of miR96 Mimic or SB216641

(A–F) RV inotropy as measured by dp/dt max (A and B), lusitropy as measured by dp/dt min (C and D), and RV load as measured by arterial elastance (E and F). Nx Veh, normoxic vehicle; Su/Hx Veh, sugen/hypoxic vehicle; Su/Hx SB216641, sugen/hypoxic group treated with SB216641; Nx scramble, normoxic scramble-sequence-treated group; Nx Mimic, normoxic group treated with miR96 mimic; Su/Hx Scramble: sugen/hypoxic group treated with scramble sequence; Su/Hx Mimic: sugen/hypoxic group treated with miR96 mimic. Error bars indicate mean ± SEM. One-way ANOVA with post hoc Tukey test was used to assess statistical significance. ⋆$p < 0.05$; ⋆⋆$p < 0.01$; ⋆⋆⋆$p < 0.001$. n = 4–8.

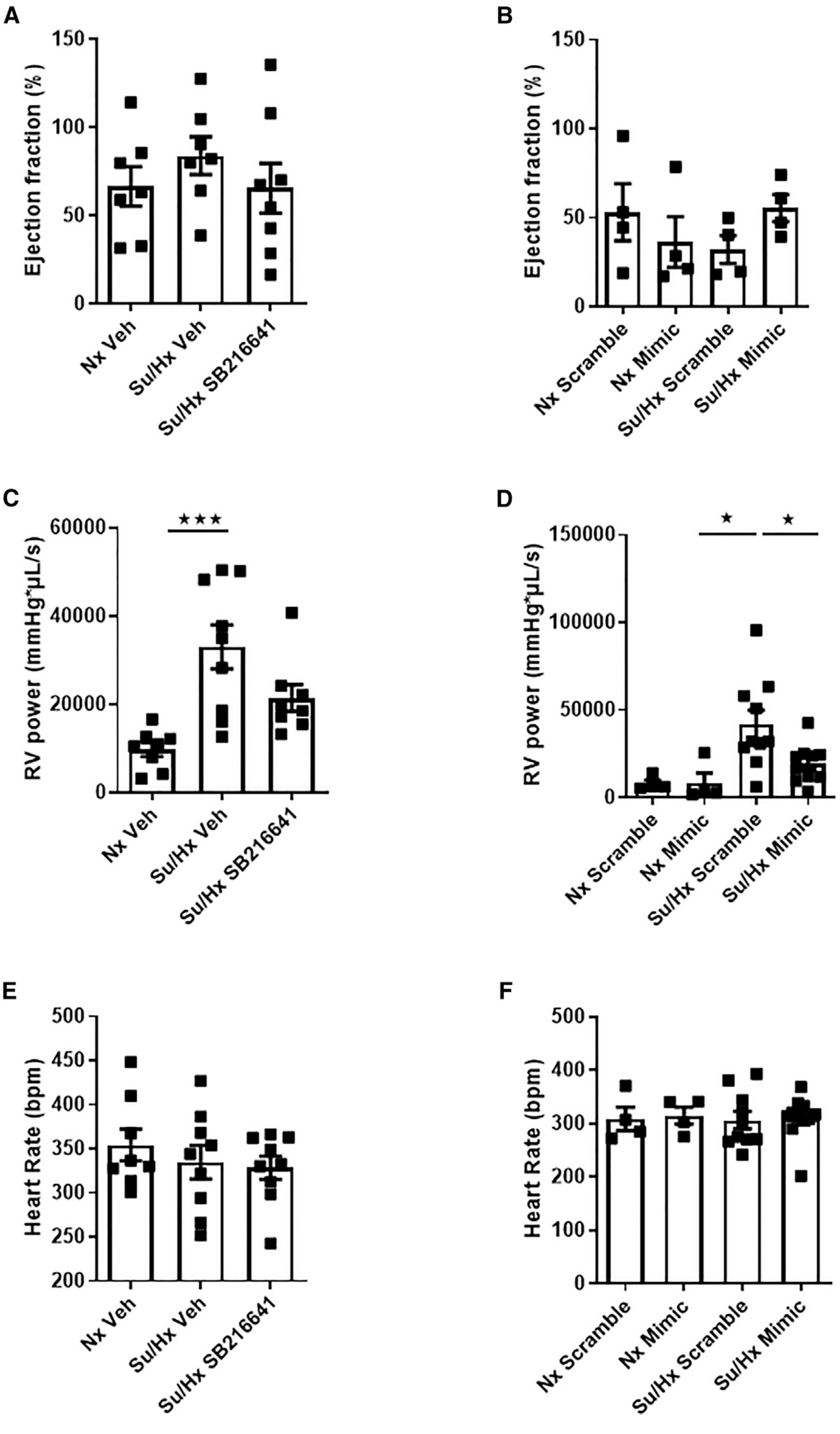

Figure 5. Hemodynamic Assessment of RV Function Su/Hx Rat Model in the Presence of miR96 Mimic or SB216641

(A–F) Ejection fraction (A and B), RV loop power (C and D), and heart rate (E and F) in Nx Veh and Su/Hx and SB216641/Veh-treated rats or in normoxic scramble and normoxic miR96 mimic-treated versus Su/Hx scramble sequence-treated and Su/Hx miR96-mimic-treated rats. Error bars indicate mean ± SEM. One-way ANOVA with post hoc Tukey test was used to assess statistical significance. ★$p < 0.05$; ★★★$p < 0.001$. n = 4–8.

so there was no requirement to investigate this further in the present study. Through the 5HT1BR, serotonin induces Src-related, kinase-regulated, Nox1-induced reactive oxygen species (ROS) and Nrf-2 dysregulation. This contributes to increased post-translational oxidative modification of proteins and activation of redox-sensitive signaling pathways in hPASMCs and is associated with the mitogenic responses in hPASMCs.[19] Rho A/Rho kinase also participate in 5HT1BR-mediated mitogenesis through an effect on cytoplasmic-to-nuclear translocation of ERK1/ERK2.[30] Serotonin can transactivate the serine kinase receptor BMPR1A to activate Smads 1/5/8 via Rho and Rho kinase transglutaminase. This also contributes to serotonin-induced PASMC proliferation via AKT signaling.[31] In addition, we have shown that 5HT1BR activity is enhanced by increased vascular tone through pharmacological synergy.[28]

miR96 increased lung BMPR2 protein expression in the sugen/hypoxic rats. BMPR2 expression and activity are crucial in the pulmonary vasculature and mutations in *BMPR2* underlie the vast majority of heritable forms of PAH.[32,33] Furthermore, many idiopathic cases of PAH are a result of dysfunctional BMPR2 signaling.[34] Therefore, increased BMPR2 signaling may have contributed to the therapeutic effects of miR96. We have also previously shown that there is increased 5HT1BR gene and protein in *BMPR2* mutation knockin mice, suggesting an inverse relationship between 5HT1BR expression and BMPR2 signaling.[12]

It was important to investigate any off-target effects of i.t.-administered miR96. miR96 had no effects on liver or kidney function. Mast cells are markers for allergic and inflammatory responses.[35] The absence of inflammatory mRNA markers or mast cell numbers in the lungs after miR96 indicates that miR96 itself does not cause any off-target inflammation that may be detrimental to the health of the animal. This suggests that direct delivery of the miR96 mimic to the lungs may prevent excessive off-target effects that may be produced by intravenous delivery or other approaches.

Other limitations of this study include the necessity to study open-chested anesthetized animals and financial constraints limiting our

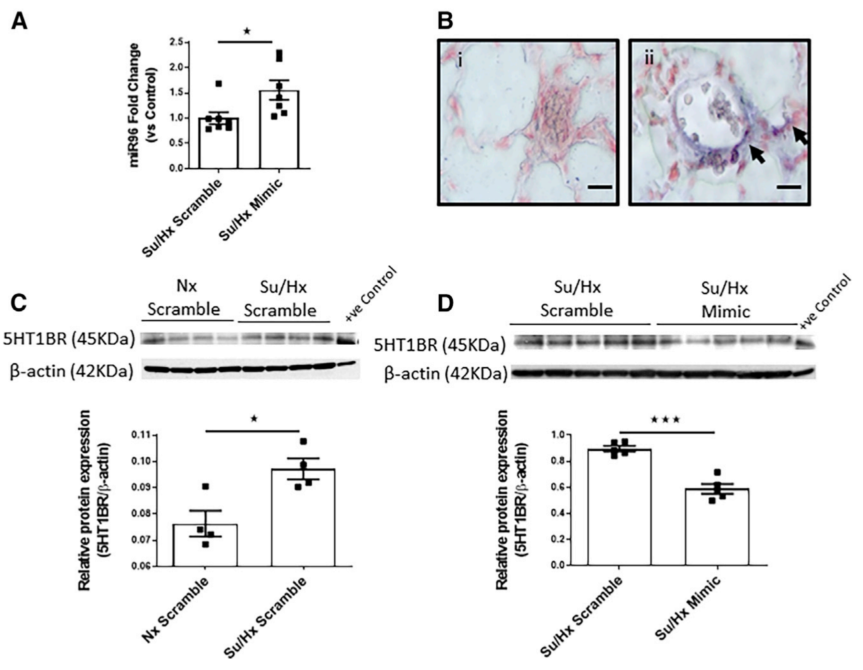

Figure 6. Expression of miR96 Mimic in the Lung and Pulmonary Artery

(A) miR96 expression in the lungs of Su/Hx scramble sequence and Su/Hx miR96 mimic-treated rats. (B) miR96 expression in the pulmonary artery of Su/Hx scramble sequence (i) and Su/Hx miR96-mimic-treated rats (ii); miR96 (purple) is indicated by arrows. (C and D) 5HT1BR protein expression in whole lung tissue from normoxic scramble sequence (C) and Su/Hx miR96 mimic-treated rats (D). Data are expressed as the ratio of expression (target/loading control). Error bars indicate mean ± SEM. One-way ANOVA with post hoc Tukey test was used to assess statistical significance. *$p < 0.05$; ***$p < 0.001$. Scale bars, 20 μm.

ability to study the effects of dosing with miR96 over a longer period of time. Our whole-lung expression approach, required to harvest sufficient tissue, did not allow us to identify potential cellular compartmentalization of the miR96.

In conclusion, our studies have shown that, in the sugen/hypoxic rat model, miR96 delivered directly to the lungs is therapeutic and tolerable, with no observed side effects in the kidney, in the liver, or on inflammatory markers. We demonstrate that miR96 may exert therapeutic effects through decreased 5HT1BR and signaling, leading to decreased progression of pulmonary vascular remodeling.

MATERIALS AND METHODS

In Vivo

The procedures conform to the guidelines from Directive 2010/63/EU of the European Parliament on the protection of animals used for scientific purposes. All animal procedures performed were also approved by the UK Home Office according to regulations regarding experiments with animals in the United Kingdom (ASPA 1986) and therefore approved by local ethical review (#P15C58D3F).

Female Sprague-Dawley rats (Charles River Laboratories), 125–150 g, were dosed subcutaneously with 30 mg/kg sugen and exposed to 21 days of hypobaric hypoxia (550 mbar) modified from methods described previously.[36,37] Rats were then maintained in normoxia for 21 days. Control rats were maintained in normoxic conditions. Treatment with drug or miR96 started immediately after the hypoxic/control period (day 21). Rats were treated for 3 weeks as follows: (1) ddH_2O/vehicle or 5HT1BR antagonist SB216641 (dosed orally, daily [7.5 mg/kg]); (2) negative scramble sequence control or miR96 mimic (Applied Biosystems) delivered with Invivofectamine (Thermo Fisher Scientific, Winsford, UK) prepared in accordance with the manufacturer's instructions (dosed weekly intra-tracheally [100 μL; 50 μg per rat per week for 3 weeks]). Rat cardiac hemodynamics were measured using the Millar Pressure-Volume (PV) Loop System after the 3 weeks of interventions.[37] Anesthesia was delivered via a facemask using 3% isofluorane with an air/oxygen mixture at a flow rate of 1 L/min. Depth of anesthesia was continuously monitored throughout to ensure that the animal remained unconscious and insentient. This was assessed by pedal withdrawal reflex and visual monitoring of respiration rate and rhythm. Euthanasia was carried out under terminal anesthesia by exsanguination via cardiac puncture before heart and lungs were removed.

An additional group of rats were left for 3 weeks after the last dose of miR96 or scramble sequence control before their hemodynamics were measured. This was in order to test the longer term effects of miR96. See the Supplemental Information for further details.

RVH

RVH was assessed as the right ventricle weight expressed as a ratio to the left ventricle plus septum weight (Fulton index ratio).[37] See the Supplemental Information for further details.

qRT-PCR

Briefly, all RNA was extracted using the miRNeasy Mini Kit following the manufacturer's protocol (QIAGEN, Manchester, UK). RNA was then reverse transcribed to cDNA before being probed with specific TaqMan primer-probe sets (Thermo Fisher Scientific, Winsford, UK).[38] See the Supplemental Information for further details.

Western Blotting

Protein expression was assessed by immunoblotting in whole lung tissue.[22] See the Supplemental Information for further details.

Creatinine Assay

Plasma and urine creatinine levels were measured using the 96-well colorimetric Creatinine Assay Kit (ab65340, Abcam, Cambridge, UK) following the manufacturer's protocol.

ALT Assay

Rat plasma ALT levels were assessed using the colorimetric 96-well Rat ALT ELISA Kit (ab234579, Abcam, Cambridge, UK).

Histology

Pulmonary artery remodeling and lung mast cell staining were assessed using histological techniques that have been described previously.[39] See the Supplemental Information for further details.

In Situ Hybridization

Localization of miR96 within the lung was assessed using *in situ* hybridization as described previously[12,40] and in the Supplemental Information.

Statistical Analysis

Either a one-way ANOVA with Tukey's post hoc test was used to determine significance of variances (as indicated in the figure legend of each figure), or unpaired Student's t test was used where appropriate. All graphs and statistical analyses were produced and performed using GraphPad Prism v.5. All data are presented as mean ± SEM, and $p < 0.05$ was considered statistically significant.

SUPPLEMENTAL INFORMATION

Supplemental Information can be found online at https://doi.org/10.1016/j.omtn.2020.09.002.

AUTHOR CONTRIBUTIONS

Conceptualization, M.R.M. and C.K.D.; Investigation, C.K.D., S.F., D.H., M.N., R.L.O., and H.L.; Formal Analysis, C.K.D., M.N., H.L., R.L.O., and N.D.; Writing and Formatting, C.K.D., N.D., and M.R.M.; Funding Acquisition, M.R.M.

CONFLICTS OF INTEREST

The authors declare no competing interests.

ACKNOWLEDGMENTS

This study is supported by an Investigator Sponsored Study from United Therapeutics (70681/2) and the Medical Research Council (grant number MR/N/011112/1).

REFERENCES

1. Humbert, M., Morrell, N.W., Archer, S.L., Stenmark, K.R., MacLean, M.R., Lang, I.M., Christman, B.W., Weir, E.K., Eickelberg, O., Voelkel, N.F., and Rabinovitch, M. (2004). Cellular and molecular pathobiology of pulmonary arterial hypertension. J. Am. Coll. Cardiol. *43* (12, Suppl S), 13S–24S.
2. MacLean, M.M.R. (2018). The serotonin hypothesis in pulmonary hypertension revisited: targets for novel therapies (2017 Grover Conference Series). Pulm. Circ. *8*, 2045894018759125.
3. Dempsie, Y., and MacLean, M.R. (2008). Pulmonary hypertension: therapeutic targets within the serotonin system. Br. J. Pharmacol. *155*, 455–462.
4. Eddahibi, S., Humbert, M., Fadel, E., Raffestin, B., Darmon, M., Capron, F., Simonneau, G., Dartevelle, P., Hamon, M., and Adnot, S. (2001). Serotonin transporter overexpression is responsible for pulmonary artery smooth muscle hyperplasia in primary pulmonary hypertension. J. Clin. Invest. *108*, 1141–1150.
5. Morecroft, I., Dempsie, Y., Bader, M., Walther, D.J., Kotnik, K., Loughlin, L., Nilsen, M., and MacLean, M.R. (2007). Effect of tryptophan hydroxylase 1 deficiency on the development of hypoxia-induced pulmonary hypertension. Hypertension *49*, 232–236.
6. Izikki, M., Hanoun, N., Marcos, E., Savale, L., Barlier-Mur, A.M., Saurini, F., Eddahibi, S., Hamon, M., and Adnot, S. (2007). Tryptophan Hydroxylase 1 (Tph1) Knock-Out and Tph2 Polymorphism: Effects on Hypoxic Pulmonary Hypertension in Mice. Am. J. Physiol. Lung Cell. Mol. Physiol. *293*, 1045–1052.
7. Ciuclan, L., Hussey, M.J., Burton, V., Good, R., Duggan, N., Beach, S., Jones, P., Fox, R., Clay, I., Bonneau, O., et al. (2012). Imatinib attenuates hypoxia-induced PAH pathology via reduction in 5-HT through inhibition of TPH1 expression. Am. J. Respir. Crit. Care Med. *187*, 78–89.
8. Aiello, R.J., Bourassa, P.A., Zhang, Q., Dubins, J., Goldberg, D.R., De Lombaert, S., Humbert, M., Guignabert, C., Cavasin, M.A., McKinsey, T.A., and Paralkar, V. (2017). Tryptophan hydroxylase 1 Inhibition Impacts Pulmonary Vascular Remodeling in Two Rat Models of Pulmonary Hypertension. J. Pharmacol. Exp. Ther. *360*, 267–279.
9. MacLean, M.R., Deuchar, G.A., Hicks, M.N., Morecroft, I., Shen, S., Sheward, J., Colston, J., Loughlin, L., Nilsen, M., Dempsie, Y., and Harmar, A. (2004). Overexpression of the 5-hydroxytryptamine transporter gene: effect on pulmonary hemodynamics and hypoxia-induced pulmonary hypertension. Circulation *109*, 2150–2155.
10. Morecroft, I., Heeley, R.P., Prentice, H.M., Kirk, A., and MacLean, M.R. (1999). 5-hydroxytryptamine receptors mediating contraction in human small muscular pulmonary arteries: importance of the 5-HT1B receptor. Br. J. Pharmacol. *128*, 730–734.
11. MacLean, M.R., Clayton, R.A., Templeton, A.G.B., and Morecroft, I. (1996). Evidence for 5-HT1-like receptor-mediated vasoconstriction in human pulmonary artery. Br. J. Pharmacol. *119*, 277–282.
12. Wallace, E., Morrell, N.W., Yang, X.D., Long, L., Stevens, H., Nilsen, M., Loughlin, L., Mair, K.M., Baker, A.H., and MacLean, M.R. (2015). A Sex-Specific MicroRNA-96/5-Hydroxytryptamine 1B Axis Influences Development of Pulmonary Hypertension. Am. J. Respir. Crit. Care Med. *191*, 1432–1442.
13. Keegan, A., Morecroft, I., Smillie, D., Hicks, M.N., and MacLean, M.R. (2001). Contribution of the 5-HT(1B) receptor to hypoxia-induced pulmonary hypertension: converging evidence using 5-HT(1B)-receptor knockout mice and the 5-HT(1B/1D)-receptor antagonist GR127935. Circ. Res. *89*, 1231–1239.
14. Zhou, G., Chen, T., and Raj, J.U. (2015). MicroRNAs in pulmonary arterial hypertension. Am. J. Respir. Cell Mol. Biol. *52*, 139–151.
15. Millan, M.J. (2011). MicroRNA in the regulation and expression of serotonergic transmission in the brain and other tissues. Curr. Opin. Pharmacol. *11*, 11–22.
16. Liao, X.J., Mao, W.M., Wang, Q., Yang, G.G., Wu, W.J., and Shao, S.X. (2016). MicroRNA-24 inhibits serotonin reuptake transporter expression and aggravates irritable bowel syndrome. Biochem. Biophys. Res. Commun. *469*, 288–293.
17. Lee, S.L., Wang, W.W., Finlay, G.A., and Fanburg, B.L. (1999). Serotonin stimulates mitogen-activated protein kinase activity through the formation of superoxide anion. Am. J. Physiol. *277*, L282–L291.
18. Liu, Y., Ren, W., Warburton, R., Toksoz, D., and Fanburg, B.L. (2009). Serotonin induces Rho/ROCK-dependent activation of Smads 1/5/8 in pulmonary artery smooth muscle cells. FASEB J. *23*, 2299–2306.
19. Hood, K.Y., Mair, K.M., Harvey, A.P., Montezano, A.C., Touyz, R.M., and MacLean, M.R. (2017). Serotonin Signaling Through the 5-HT_{1B} Receptor and NADPH Oxidase 1 in Pulmonary Arterial Hypertension. Arterioscler. Thromb. Vasc. Biol. *37*, 1361–1370.
20. Abe, K., Toba, M., Alzoubi, A., Ito, M., Fagan, K.A., Cool, C.D., Voelkel, N.F., McMurtry, I.F., and Oka, M. (2010). Formation of plexiform lesions in experimental severe pulmonary arterial hypertension. Circulation *121*, 2747–2754.
21. Chrystyn, H. (2001). Methods to identify drug deposition in the lungs following inhalation. Br. J. Clin. Pharmacol. *51*, 289–299.

22. Falcão, L.M., Pinto, F., Ravara, L., and van Zwieten, P.A. (2004). BNP and ANP as diagnostic and predictive markers in heart failure with left ventricular systolic dysfunction. J. Renin Angiotensin Aldosterone Syst. *5*, 121–129.

23. Leach, E., Howard, P., and Barer, G.R. (1977). Resolution of hypoxic changes in the heart and pulmonary arterioles of rats during intermittent correction of hypoxia. Clin. Sci. Mol. Med. *52*, 153–162.

24. Liu, A., Schreier, D., Tian, L., Eickhoff, J.C., Wang, Z., Hacker, T.A., and Chesler, N.C. (2014). Direct and indirect protection of right ventricular function by estrogen in an experimental model of pulmonary arterial hypertension. Am. J. Physiol. Heart Circ. Physiol. *307*, H273–H283.

25. Morecroft, I., Loughlin, L., Nilsen, M., Colston, J., Dempsie, Y., Sheward, J., Harmar, A., and MacLean, M.R. (2005). Functional interactions between 5-hydroxytryptamine receptors and the serotonin transporter in pulmonary arteries. J. Pharmacol. Exp. Ther. *313*, 539–548.

26. Morecroft, I., Pang, L., Baranowska, M., Nilsen, M., Loughlin, L., Dempsie, Y., Millet, C., and MacLean, M.R. (2010). In vivo effects of a combined 5-HT1B receptor/SERT antagonist in experimental pulmonary hypertension. Cardiovasc. Res. *85*, 593–603.

27. Dickenson, J.M., and Hill, S.J. (1998). Human 5-HT1B receptor stimulated inositol phospholipid hydrolysis in CHO cells: synergy with Gq-coupled receptors. Eur. J. Pharmacol. *348*, 279–285.

28. MacLean, M.R. (1999). Pulmonary hypertension, anorexigens and 5-HT: pharmacological synergism in action? Trends Pharmacol. Sci. *20*, 490–495.

29. White, K., Dempsie, Y., Nilsen, M., Wright, A.F., Loughlin, L., and MacLean, M.R. (2011). The serotonin transporter, gender, and 17β oestradiol in the development of pulmonary arterial hypertension. Cardiovasc. Res. *90*, 373–382.

30. Liu, Y., Suzuki, Y.J., Day, R.M., and Fanburg, B.L. (2004). Rho kinase-induced nuclear translocation of ERK1/ERK2 in smooth muscle cell mitogenesis caused by serotonin. Circ. Res. *95*, 579–586.

31. Penumatsa, K., Abualkhair, S., Wei, L., Warburton, R., Preston, I., Hill, N.S., Watts, S.W., Fanburg, B.L., and Toksoz, D. (2014). Tissue transglutaminase promotes serotonin-induced AKT signaling and mitogenesis in pulmonary vascular smooth muscle cells. Cell. Signal. *26*, 2818–2825.

32. Lane, K.B., Machado, R.D., Pauciulo, M.W., Thomson, J.R., Phillips, J.A., 3rd, Loyd, J.E., Nichols, W.C., and Trembath, R.C.; International PPH Consortium (2000). Heterozygous germline mutations in BMPR2, encoding a TGF-beta receptor, cause familial primary pulmonary hypertension. Nat. Genet. *26*, 81–84.

33. Hara, H., Takeda, N., Morita, H., Hatano, M., Amiya, E., Maki, H., Minatsuki, S., Taki, M., Shiraishi, Y., Fujiwara, T., et al. (2017). Three novel *BMPR2* mutations associated with advanced pulmonary arterial hypertension. Hum. Genome Var. *4*, 17010.

34. Evans, J.D.W., Girerd, B., Montani, D., Wang, X.J., Galiè, N., Austin, E.D., Elliott, G., Asano, K., Grünig, E., Yan, Y., et al. (2016). BMPR2 mutations and survival in pulmonary arterial hypertension: an individual participant data meta-analysis. Lancet Respir. Med. *4*, 129–137.

35. Galli, S.J., Gaudenzio, N., and Tsai, M. (2020). Mast Cells in Inflammation and Disease: Recent Progress and Ongoing Concerns. Annu. Rev. Immunol. *38*, 49–77.

36. Dean, A., Nilsen, M., Loughlin, L., Salt, I.P., and MacLean, M.R. (2016). Metformin Reverses Development of Pulmonary Hypertension via Aromatase Inhibition. Hypertension *68*, 446–454.

37. Docherty, C.K., Nilsen, M., and MacLean, M.R. (2019). Influence of 2-Methoxyestradiol and Sex on Hypoxia-Induced Pulmonary Hypertension and Hypoxia-Inducible Factor-1-α. J. Am. Heart Assoc. *8*, e011628.

38. Mair, K.M., Yang, X.D., Long, L., White, K., Wallace, E., Ewart, M.A., Docherty, C.K., Morrell, N.W., and MacLean, M.R. (2015). Sex affects bone morphogenetic protein type II receptor signaling in pulmonary artery smooth muscle cells. Am. J. Respir. Crit. Care Med. *191*, 693–703.

39. Dean, A., Gregorc, T., Docherty, C.K., Harvey, K.Y., Nilsen, M., Morrell, N.W., and MacLean, M.R. (2018). Role of the Aryl Hydrocarbon Receptor in Sugen 5416-induced Experimental Pulmonary Hypertension. Am. J. Respir. Cell Mol. Biol. *58*, 320–330.

40. Caruso, P., Dempsie, Y., Stevens, H.C., McDonald, R.A., Long, L., Lu, R., White, K., Mair, K.M., McClure, J.D., Southwood, M., et al. (2012). A Role for miR-145 in Pulmonary Arterial Hypertension/Novelty and Significance. Circ. Res. *111*, 290–300.

OMTN, Volume 22

Supplemental Information

Direct Delivery of MicroRNA96 to the Lungs Reduces Progression of Sugen/Hypoxia-Induced Pulmonary Hypertension in the Rat

Craig K. Docherty, Nina Denver, Simon Fisher, Margaret Nilsen, Dianne Hillyard, Rebecca L. Openshaw, Hicham Labazi, and Margaret R. MacLean

Online Data Supplement

Materials and methods

In vivo

A microsprayer 1A-1B (Penn Century) and gas-tight SGE syringe (Hamilton syringe) was primed with PBS prior to procedure and then sterilized with ethanol. Female Sprague Dawley rats (Charles River), 125-150g, were induced into deep anesthesia using using 5% Isofluorane with an air/oxygen mixture at a flow rate of 1L/min into an induction chamber for 5 to 7 minutes to ensure deep anaesthesia. Animals are assessed by pedal withdrawal reflex and monitoring of respiration rate and rhythm. After suspending by incisors at a 45 degree angle, blunt forceps were used to position the tongue so it did not obscure the trachea. The microsprayer was loaded with 100µL of miR96 mimic or scramble sequence and slowly inserted past the epiglottal space and vocal cords to the region directly above the bifurcation of the bronchi, visualized using a light source. The sprayer plunger was quickly and forcefully depressed and held in place for 10 seconds. Animals were then held upright for a further 10-15 seconds before recovery in a warm cage. Animals were closely observed until fully recovered and placed back into their original cage.

Hemodynamic measurements

Rats were anesthetized in 3-4% (v/v) isoflurane, the level of anesthesia was assessed by absence of pedal reflex to toe pinch. The rats were placed on a heating pad, where the rat’s front paws and one distal paw were taped down. Following a neck midline incision, the pretracheal muscles were pulled apart gently with forceps and dissected the around the trachea. A surgical silk suture was placed underneath the trachea, a small cut onto the surface of the trachea was made into which the tracheotomy cannula was inserted and secured with the silk suture. Then, the rats were immediately transferred to a ventilator where they were maintained at approximately 1.5-2% (v/v)

isoflurane supplemented with a constant flow of medical oxygen (0.5L/min). Body temperature (37 °C) was monitored by a probe that was inserted into the rat's rectum. Prior to performing open chest surgery, the level of anesthesia of was assessed by absence reflex to toe pinch. Respiratory rate and tidal volume were adjusted for each individual rat based on its weight. Following an incision made over the xyphoid process, a cut was made through the chest wall moving laterally on both sides until the diaphragm is clearly visible, then a thoracotomy was performed to expose the chest cavity. Following a gentle removal of the pericardium from the heart with forceps, a needle was used make a hole into the right ventricle, through which the SPR-869 catheter (A Miller (UK) SPR-869 catheter, with an effective length of 12.5 cm and 4 platinum electrodes, and a pressure sensor centred between pairs of electrodes, was used as per the manufacturer's instructions) was inserted into the right ventricle allowing right ventricular systolic pressure (RVSP) measurements which were recorded using PowerLab data acquisition system with its corresponding software (LabChart Pro). Other cardiac parameters were also measured using the pressure-volume (PV) loop system which were calculated automatically using the Labchart 8 PV loop module software; maximum derivative of pressure/ derivative of time (dp/dt max) which is an indirect measure of cardiac contractility (inotropy), minimum derivative of pressure/ derivative of time (dp/dt min) is an indirect measure of cardiac relaxation (lusitropy), arterial elastance (RV pressure at end of systole/ (max volume - min volume)) is a measure of RV load, ejection fraction which is the volumetric fraction of fluid ejected during each heartbeat, stroke volume which is the maximum volume minus the minimum volume during a PV loop/cardiac cycle, stroke work (area enclosed by the PV loop) and RV power is a measure of ventricular efficiency/workload using maximum values of pressure and volume. All analysis was carried out blind and by licensed, experienced researchers. For vena cava occlusions (to examone cardiac response over a range of preloads and can be obtained with IVC constriction?. a suture was placed beneath the inferior vena cava (VC), at the end of the normal PV

loop recording, using a needle clamp the suture was gently and slowly raised over 1-2 sec.

Histopathology

Pulmonary vascular remodelling was assessed using 5µm sections cut and stained (4-6 sections per lung) using Millers elastin/Picro Sirius Red for identification of vascular remodelling, characterized by a distinctive double elastic lamina. The total number of remodelled vessels (≥80 per lung) was expressed over the total number of vessels present in a lung section in a blinded fashion. Occluded vessels/lesions were counted using lung sections stained with Von Willebrand staining. Mast cells were identified in lung sections stained with toluidine blue and counted per lung section. Images were acquired using a Zeiss Axiocam 305 color (Carl Zeiss Microscopy GmbH, Germany).

MiR96 in-situ hybridization

5µm lung tissue sections were deparaffinised in xylene, rehydrated by passing through graded ethanol (100%, 90%, 70%) then washed for 5 minutes in sterile PBS. Sections were treated with 400µg/ml proteinase K (Invitrogen, 25530049) at 37°C for 10 minutes followed by 2 x 5-minute washes in sterile PBS and dehydrated: 2 x 1 minute washes in 70%, 90% and 100% ethanol. Sections were then air dried for 15 minutes. DIG labelled probes complementary to miR96 (Qiagen) and U6 (Exiqon) were diluted in hybridisation buffer (Sigma, H7782) and hybridised at 52°C for 1-2 hours in a humidified chamber. This was followed by stringency washes of 5 x saline – sodium citrate buffer (SSC) at room temperature, 5 x SSC, 1 x SSC and 2 x SSC at 52°C, then 2 x SSC at room temperature. Sections were then washed in PBS-T before blocking (blocking reagent, Roche 11175041910, in maleic acid buffer) for 15 minutes at room

temperature and sections were incubated with 1:500 alkaline phosphatase AP conjugated antibody for DIG (Roche, 11175041910) in blocking solution. Sections were washed 2 x 3 minutes in PBS-T then incubated at room temperature in NBT/BCIP colour reagent (Roche, 11175041910) in substrate solution (0.1M Tris-HCl, 0.1M NaCl, pH9.5) for 6 days, protected from light. Sections were washed 5 minutes in 0.1M Tris-HCl, 2 x 1-minute water, counterstained with nuclear fast red (1 minute at room temperature), rinsed in tap water for 10 minutes then dehydrated as before and mounted using permount mounting media (Fisher, 15820100). All reagents were made using RNAse-free/ DEPC water. Images were acquired using a Zeiss Axiocam 305 color (Carl Zeiss Microscopy GmbH, Germany).

qRT-PCR

Tissue was homogenized using a mortar and pestle and RNA extracted by placing into QIAzol solution (Qiagen, UK). Tissue was lysed using a Qiagen Tissue lyser and RNA was extracted with miRNeasy® mini kit following the manufacturers protocol (Qiagen, UK). hPASMCs were scraped and also lysed using QIAzol solution. RNA was quantified using a nanodrop (ND-1000 spectrophotometer (Thermo-Fisher, UK). RNA was reverse transcribed to cDNA using the TaqMan™ reverse transcription kit (Applied Biosystems). Semi-quantitative real-time PCR was performed using an Applied Biosystems Viia 7 real-time PCR system. Specific dual labelled TaqMan™ primer-probe sets were purchased from Thermo-Fisher, UK and are shown in Table S1. Results are expressed as a ratio to a reference gene using the $2^{-\Delta Ct}$ comparison method.

Western blotting

Whole lung rat samples were homogenized and lysed in RIPA buffer (Sigma, UK) containing HALT protease and phosphatase inhibitors (Thermo-Fisher, UK). hPASMCs were lysed in ice-cold 1% (v/v) lauryl maltoside/PBS (Abcam, UK) containing HALT protease and phosphatase inhibitors. Protein concentrations were determined using BCA assay (Thermo-Fisher, UK). 20µg of protein was loaded for whole lung lysates, for protein identification by SDS-PAGE and immunoblotting. Protein expression was quantitated in immunoblots probed the relevant antibody by overnight incubation at 4°C. Antibodies used are shown in Table S2. Membranes were then incubated with anti-rabbit or anti-mouse secondary antibodies. Immunoblots were developed using Pierce™ ECL Western Blotting Substrate (Thermo-Fisher, UK) or EMD Millipore Immobilon™ Western Chemiluminescent HRP Substrate (ECL) (Fisher Scientific) and normalized to beta actin (Sigma, A5441) or alpha tubulin (Abcam, ab4074). In some instances, blots were cut and probed for two separate antibodies. This was only carried out where proteins of interest exhibited a large difference in molecular weight and where antibodies have been validated and shown to be specific for their target. Blots, where appropriate, may be stripped and re-probed using loading controls.

Body weights

End of study body weights were determined and shown in Figure S9.

Supplemental Tables

Table S1. Probe sets used within this study and Taqman™ assay ID details.

Gene	Target	Assay ID
NPPA (ANP)	Rat	Rn00664637
NPPB (BNP)	Rat	Rn00580641
CTGF	Rat	Rn01537279
FN1	Rat	Rn00569575
COL1A1	Rat	Rn01463848
COL3A1	Rat	Rn01437681
TGFβ	Rat	Rn00572010
GATA4	Rat	Rn01530459
IL6	Rat	Rn01410330
TNFα	Rat	Rn99999017
CCL5	Rat	Rn00579590
B2M	Rat	Rn00560865
GAPDH	Rat	Rn01775763
miR-96	Rat/Human	RT000186
U87	Rat/Human	RT001712
miR-16	Rat/Human	RT000391

Table S2. Antibodies and dilutions used for Western blotting

Antibody	Source	Supplier (catalogue #)	Dilution
5HT1BR	Rabbit	Abcam (13896)	WB 1:500
BMPR2	Mouse	BD Bioscience (612292)	WB 1:500
β-actin	Mouse	Sigma (A5441)	WB 1:5000
α-tubulin	Rabbit	Abcam (ab4074)	WB 1:12000

Supplemental figures and legends

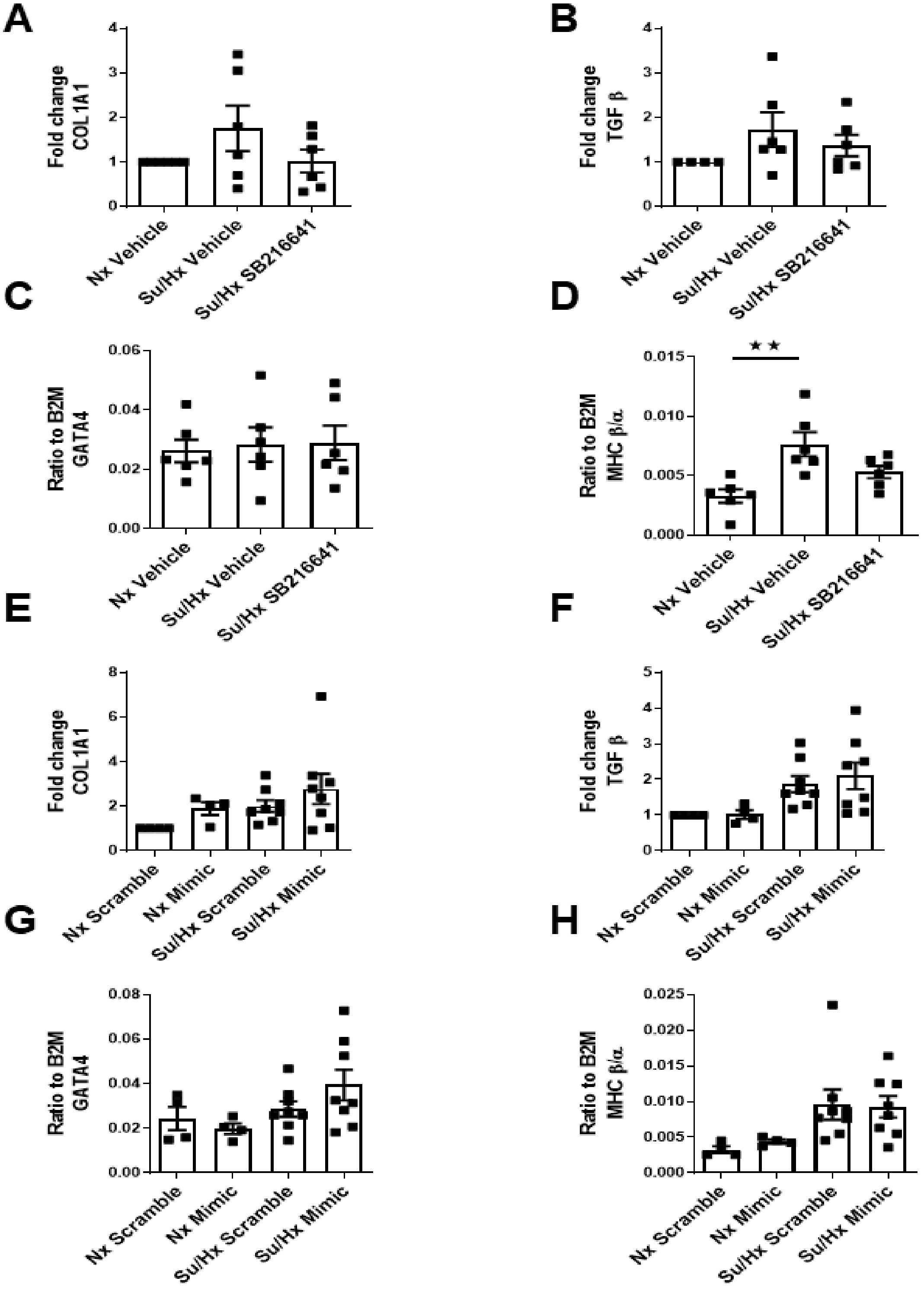

Figure S1. mRNA transcript expression of hypertrophic and fibrotic markers in the right ventricle. Collagen 1A1 (COL1A1) (A), Transforming growth factor beta

(TGFβ) (B), GATA binding protein 4 (GATA4) (C) and myosin heavy chain-β/α (β/α-MHC) (D) expression in RV tissue from normoxic (nx) vehicle and sugen/hypoxic (Su/Hx) ± SB216641 treated rats. COL1A1 (E), TGFβ (F), GATA 4 (G) and β/α-MHC (H) in normoxic miR96 mimic-treated vs sugen/hypoxic scramble sequence-treated and sugen/hypoxic miR96 mimic-treated rats. Error bars indicate mean ± SEM. One-way ANOVA with post-hoc Tukey's was used to assess statistical significance.

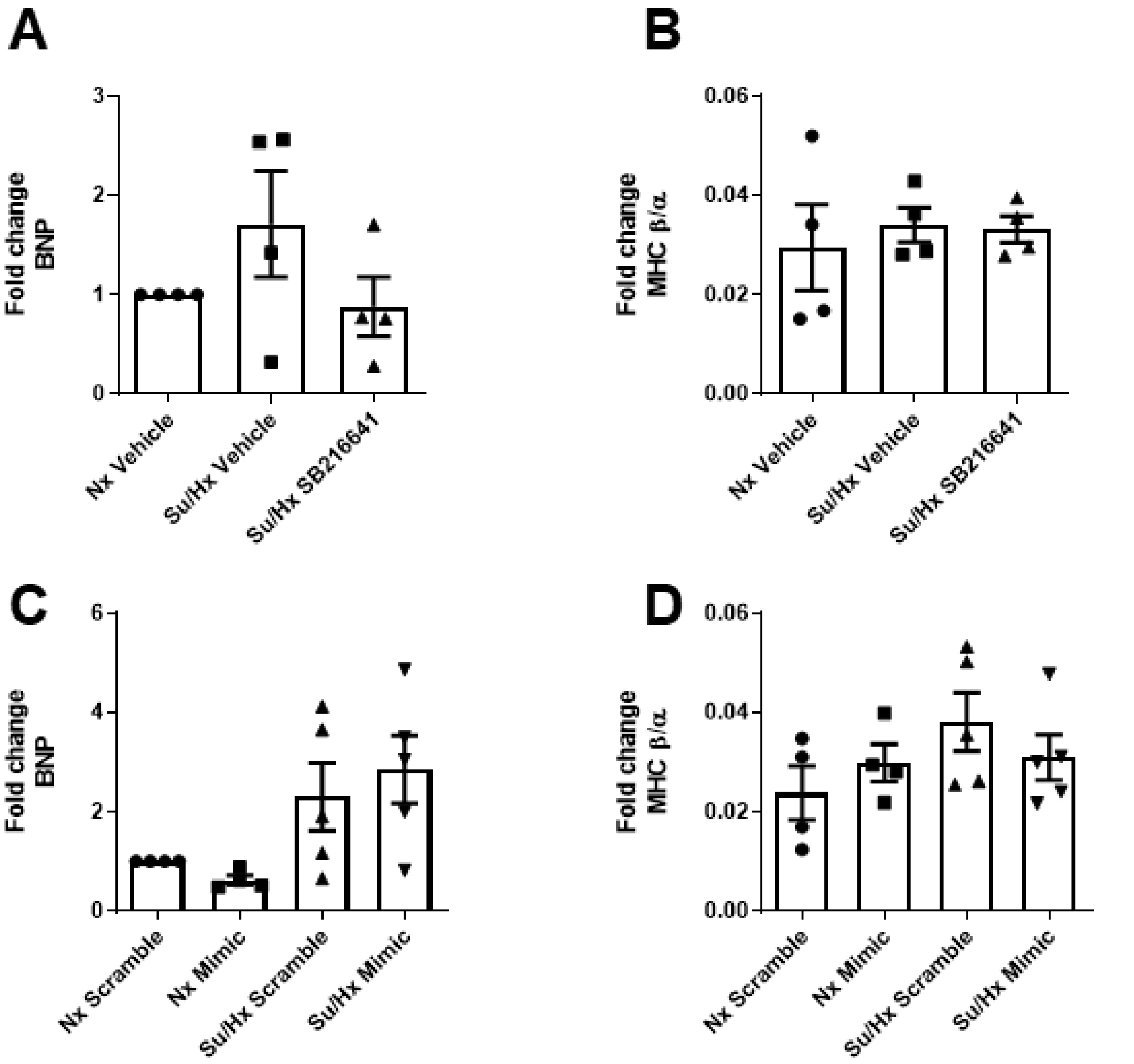

Figure S2. mRNA transcript expression of hypertrophic markers in the left ventricle. BNP (A) and β/α-MHC (B), in LV tissue from normoxic (Nx) vehicle (veh) and sugen/hypoxic (Su/Hx) and SB216641/vehicle treated rats. BNP (C) and β/α-MHC (D) expression in LV tissue from normoxic scramble sequence and normoxic miR96 mimic-treated vs sugen/hypoxic scramble sequence and sugen/hypoxic miR96-mimic treated rats. Error bars indicate mean ± SEM. One-way ANOVA with post-hoc Tukey's was used to assess statistical significance ★ = $p < 0.05$ ($n=4$-5).

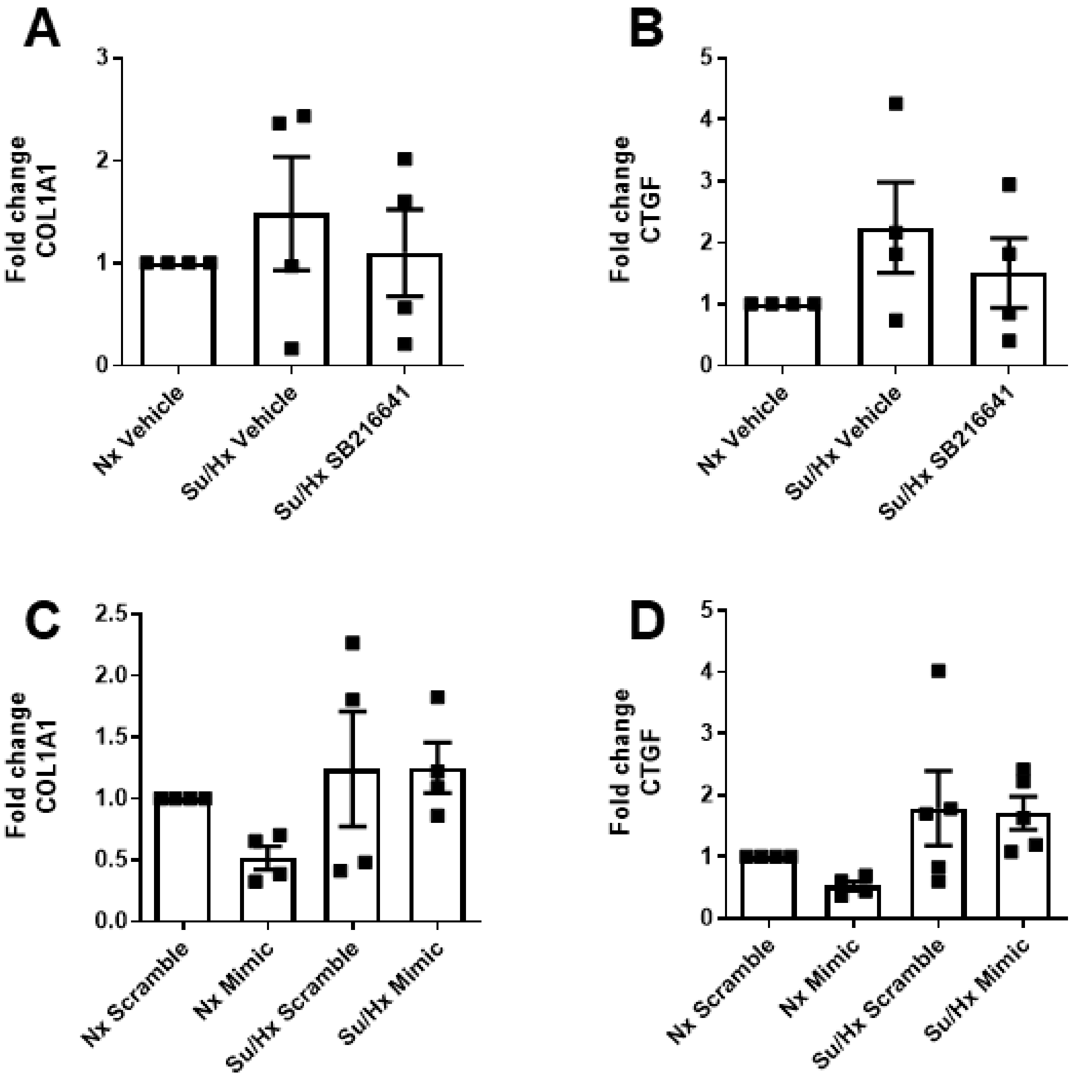

Figure S3. mRNA transcript expression of fibrotic markers in the left ventricle. COL1A1 (A) and CTGF (B) in LV tissue from normoxic (Nx) vehicle and sugen/hypoxic (Su/Hx) and SB216641/vehicle-treated rats. COL1A1 (C) and CTGF (D) in LV tissue from normoxic scramble sequence-treated and normoxic miR96 mimic –treated vs sugen/hypoxic scramble sequence-treated and sugen/hypoxic miR96 mimic-treated rats. Error bars indicate mean ± SEM. One-way ANOVA with post-hoc Tukey's was used to assess statistical significance (n=4-5).

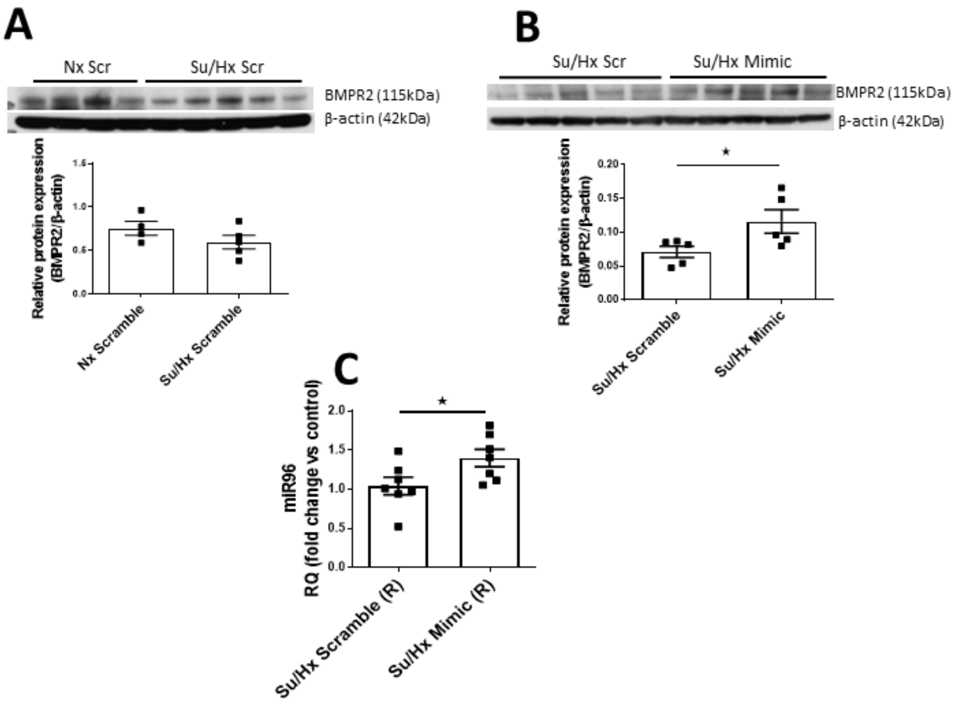

Figure S4. BMPR2 expression in Sugen-Hypoxic rat lung in presence of scramble sequence control or miR96 mimic. Representative Western Blot of BMPR2 protein expression in normoxic (Nx) scramble sequence control vs sugen/hypoxic (Su/Hx) control with densitometry analysis (A). Representative Western Blot of BMPR2 protein expression in Su/Hx scramble sequence control vs Su/Hx miR96 mimic with densitometry analysis (B). miR96 mRNA expression in the lungs of sugen/hypoxic (Su/Hx) scramble sequence and sugen/hypoxic miR96-dosed rats three weeks after last dose of miR96 (R) (C). Error bars indicate mean ± SEM. n=4-5 for each group in A, B. n=6-7 for C. Statistical significance was determined by students unpaired t-test (two-tailed). ★ $p<0.05$.

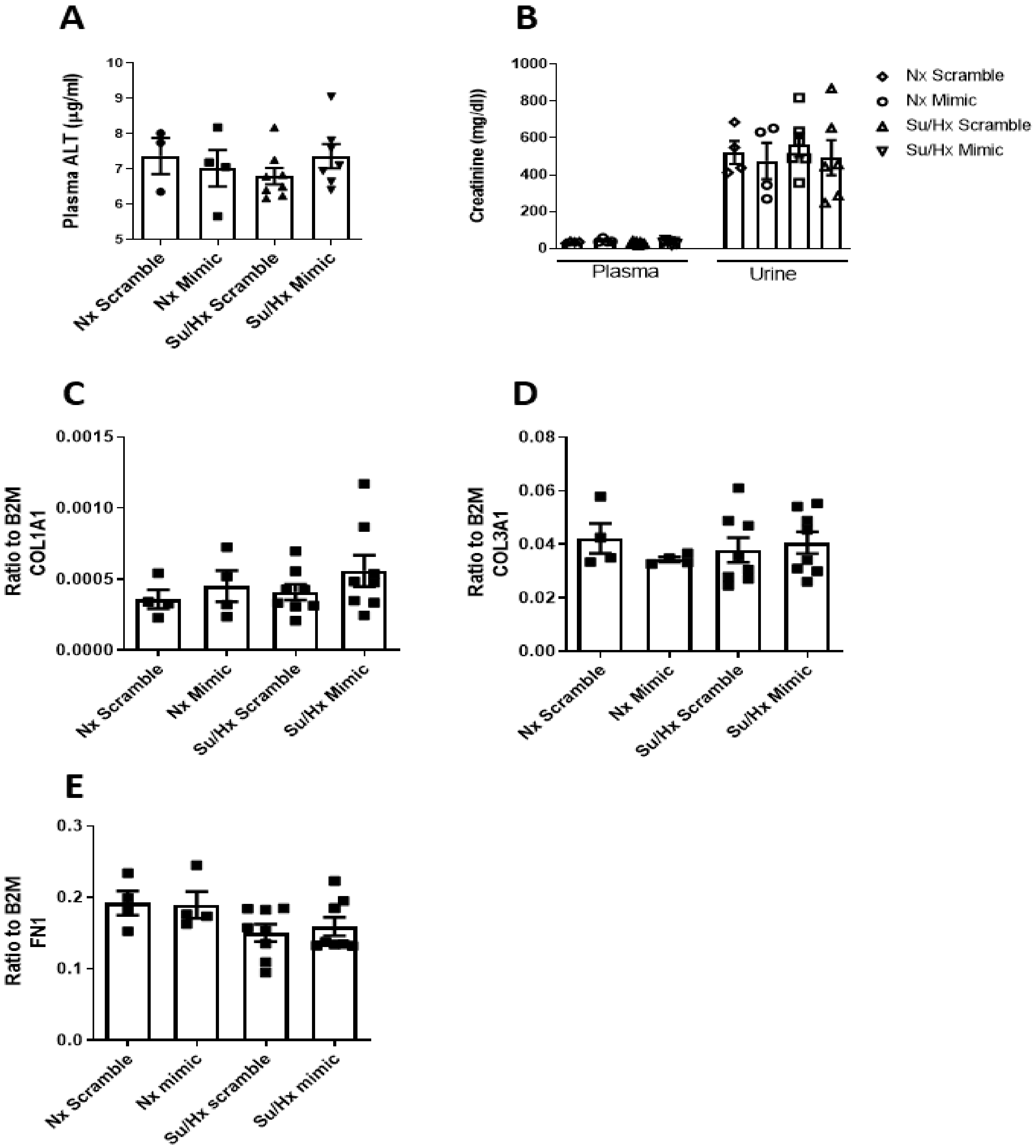

Figure S5. Effects of the miR-96 mimic on the liver and kidney. Plasma ALT levels (A) and plasma and urine creatinine levels (B) in normoxic (Nx) scramble sequence and normoxic miR96 mimic –treated vs sugen/hypoxic (Su/Hx) scramble sequence and Su/Hx miR96 mimic-treated rats. Col1a1 (C), Col3a1 (D) and FN1 (E) mRNA expression in the liver. Error bars indicate mean ± SEM. One-way ANOVA with post-hoc Tukey's was used to assess statistical significance (n=4-8).

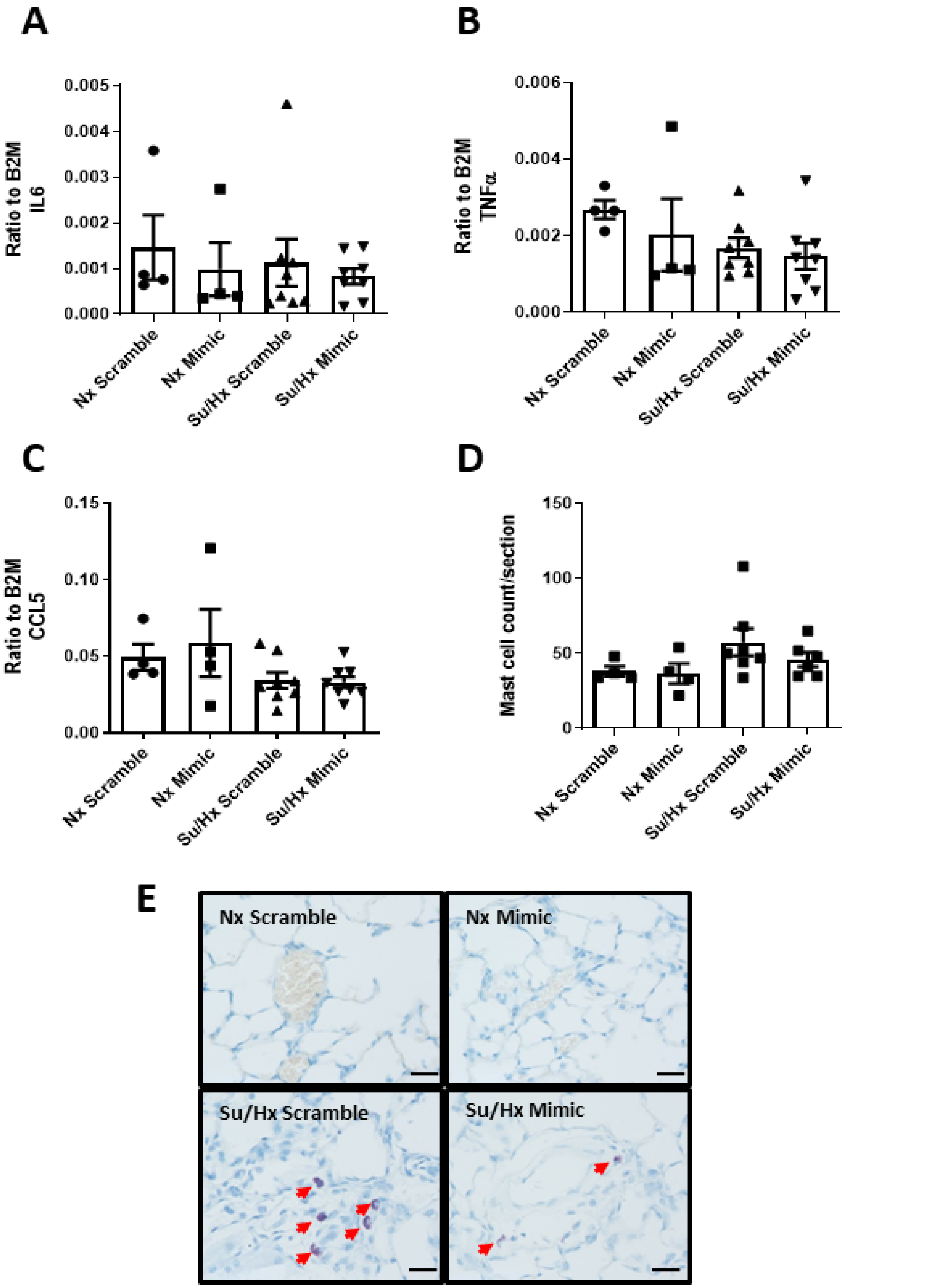

Figure S6. Effects of the miR-96 mimic on inflammation in the lung. IL6 (A), TNFα (B) and CCL5 (C) mRNA levels in lung tissue from normoxic (Nx) scramble sequence-

treated and normoxic miR96 mimic-treated vs sugen-hypoxic (Su/Hx) scramble sequence-treated and sugen-hypoxic miR96 mimic-treated rats. Mast cell number per lung section from normoxic scramble sequence-treated and normoxic miR96 mimic-treated vs sugen-hypoxic scramble sequence-treated and sugen-hypoxic miR96 mimic-treated rats (D) and representative images from each group (E). Arrows indicate mast cells. Error bars indicate mean ± SEM. One-way ANOVA with post-hoc Tukey's was used to assess statistical significance (n=4-8). Scale bars indicate 20μm.

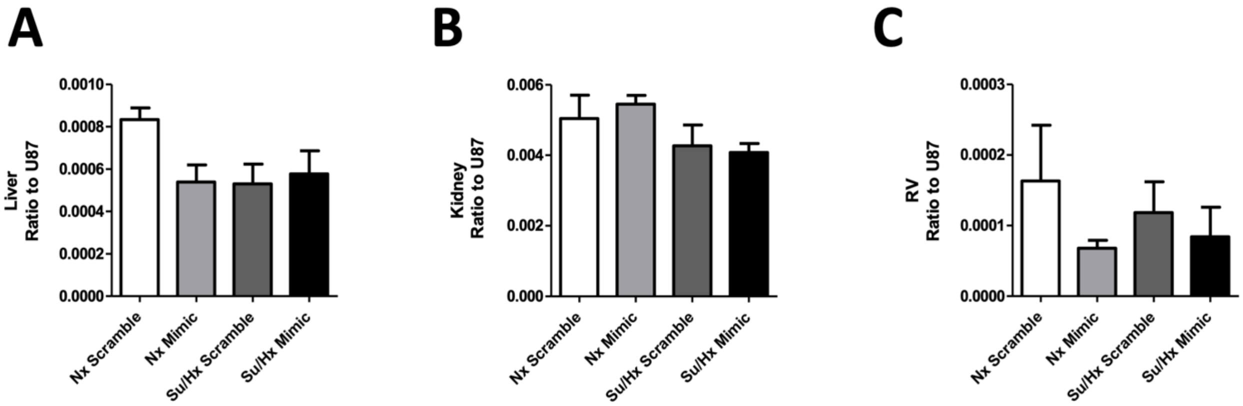

Figure S7. Expression of miR96 in liver, kidney and right ventricle (RV).

Expression of miR96 in liver (A), kidney (B), RV tissue (C) from normoxic (Nx) scramble sequence and normoxic miR96 mimic-treated vs sugen-hypoxic (Su/Hx) scramble sequence-treated and sugen-hypoxic miR96 mimic treated rats. Error bars indicate mean ± SEM. One-way ANOVA with post-hoc Tukey's was used to assess statistical significance (n=4-8).

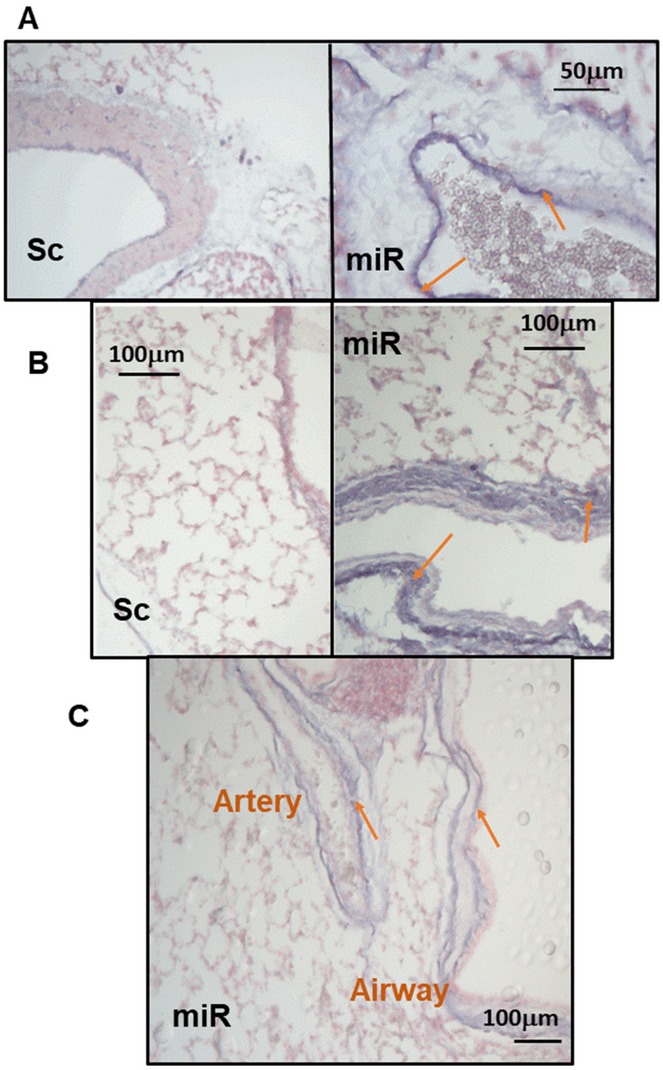

Figure S8. Expression of miR96 in large proximal pulmonary arteries and airway. MiR96 expression in large proximal pulmonary arteries (A,B) of sugen-hypoxic scramble sequence (Sc) and sugen-hypoxic miR96 mimic treated rats (miR). miR96 expression in a large proximal pulmonary artery (Artery) and adjacent airway (Airway) of sugen-hypoxic miR96 mimic treated rats (C). Positive miR96 is indicated by purple staining by arrows.

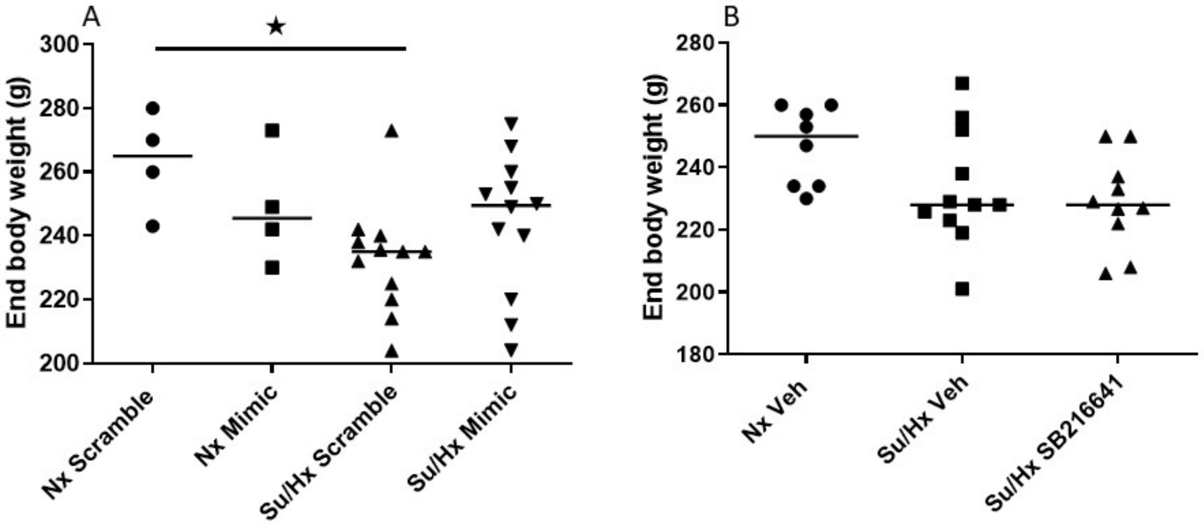

Figure S9. End of study body weights

(A) Normoxic (Nx) scramble sequence and normoxic miR96 mimic-treated vs sugen-hypoxic (Su/Hx) scramble sequence-treated and sugen-hypoxic miR96 mimic treated rats. (B) normoxic (Nx) vehicle and sugen/hypoxic (Su/Hx) and SB216641/vehicle-treated rats.
